# *MiR-199a-3p* Regulates the PTPRF/β-Catenin Axis in Hair Follicle Development: Insights into the Pathogenic Mechanism of Alopecia Areata

**DOI:** 10.3390/ijms242417632

**Published:** 2023-12-18

**Authors:** Jiankui Wang, Yuhao Ma, Tun Li, Jinnan Li, Xue Yang, Guoying Hua, Ganxian Cai, Han Zhang, Zhexi Liu, Keliang Wu, Xuemei Deng

**Affiliations:** Beijing Key Laboratory for Animal Genetic Improvement & State Key Laboratory of Animal Biotech Breeding & Key Laboratory of Animal Genetics, Breeding and Reproduction, Ministry of Agriculture, China Agricultural University, Beijing 100193, China; jiankui4955@163.com (J.W.); jzmyh2009@163.com (Y.M.); 19925489460@163.com (T.L.); ljn1374880304@outlook.com (J.L.); snow_yang2017@163.com (X.Y.); hgyyxg@163.com (G.H.); caiganxianjcau@163.com (G.C.); hanzhang@cau.edu.cn (H.Z.); bs20193040372@cau.edu.cn (Z.L.); liangkwu@cau.edu.cn (K.W.)

**Keywords:** alopecia areata, hair follicle development, Wnt/β-catenin pathway, *miR-199a-3p*, PTPRF, intracutaneous/intravenous injection

## Abstract

Alopecia areata is an autoimmune disease characterized by the immune system attacking self hair follicles, mainly in the scalp. There is no complete cure, and the pathogenesis is still not fully understood. Here, sequencing of skin tissues collected from 1-month-old coarse- and fine-wool lambs identified *miR-199a-3p* as the only small RNA significantly overexpressed in the fine-wool group, suggesting a role in hair follicle development. *MiR-199a-3p* expression was concentrated in the dermal papillae cells of sheep hair follicles, along with enhanced β-catenin expression and the inhibition of PTPRF protein expression. We also successfully constructed a mouse model of alopecia areata by intracutaneous injection with an *miR-199a-3p* antagomir. Injection of the *miR-199a-3p* agomir resulted in hair growth and earlier anagen entry. Conversely, local injection with the *miR-199a-3p* antagomir resulted in suppressed hair growth at the injection site, upregulation of immune system-related genes, and downregulation of hair follicle development-related genes. In vivo and in vitro analyses demonstrated that *miR-199a-3p* regulates hair follicle development through the PTPRF/β-catenin axis. In conclusion, a mouse model of alopecia areata was successfully established by downregulation of a small RNA, suggesting the potential value of *miR-199a-3p* in the study of alopecia diseases. The regulatory role of *miR-199a-3p* in the PTPRF/β-catenin axis was confirmed, further demonstrating the link between alopecia areata and the Wnt-signaling pathway.

## 1. Introduction

Alopecia areata is an autoimmune condition in which the body attacks its own hair follicles, characterized by localized or widespread hair loss resulting in round or patchy bald spots [1]. Alopecia is commonly observed on the scalp but can also affect the eyebrows, eyelashes, and other areas of the body with hair [2]. Although the estimated global prevalence of approximately 0.1% to 0.2% [3] represents a relatively small proportion of the population, alopecia areata can affect individuals of all ages, from children to adults [4,5]. The disease has a long history, with descriptions dating back to ancient times [6]. Alopecia has wide-ranging impacts on patients worldwide [7], significantly affecting psychological well-being and potentially causing anxiety, depression, and issues with self-image [8]. Additionally, alopecia areata presents a series of challenges on economic and social levels, including medical expenses, limited employment opportunities, and social discrimination [9]. Therefore, the global impact of alopecia areata needs to be comprehensively considered, encompassing physiological, psychological, and social aspects, to formulate better support and rehabilitation programs that provide comprehensive care and support for patients [7].

Various treatment methods can be employed based on symptoms and preferences to manage and alleviate the symptoms and improve the quality of life of patients with alopecia areata [10]. The treatment and research of alopecia areata have been widely discussed [11]. Common treatment approaches include local and systemic therapies [12]. Local treatments involve the application of topical medications such as corticosteroid creams, minoxidil, and topical immunomodulators to promote hair regrowth and reduce the affected area [3]. Conversely, systemic treatment involves the use of oral or injectable medications such as methotrexate and cyclosporine [13] to suppress abnormal immune responses and is used in patients with severe or extensive conditions [14]. Other methods, such as phototherapy, laser therapy, and oral supplementation of vitamins, have been attempted in some patients [15]. In cases of limited or no clinical efficacy of the above treatments, surgical procedures, hair transplantation, and hair follicle cell transplantation may be considered [15]. The treatment approach for alopecia areata should be tailored by physicians based on the specific circumstances of each patient to achieve optimal therapeutic outcomes [16]. However, a complete cure for alopecia areata is not yet available. Therefore, further research is needed to help unravel the specific pathogenic mechanisms of alopecia areata and provide a better theoretical basis for the treatment and intervention of this condition.

The molecular mechanisms underlying the development of alopecia areata involve multiple factors [17]. Current research indicates that both abnormal immune responses and genetic factors play important roles in the pathogenesis [18]. Immune cells such as T cells and natural killer cells may be activated and release inflammatory cytokines and cytotoxic molecules, resulting in the damage and destruction of hair follicle cells [19]. Moreover, alopecia areata is often associated with other autoimmune diseases such as thyroid disorders and rheumatoid arthritis [20]. Genetic factors are believed to play significant roles in the onset of alopecia areata [21] with certain genetic variations increasing individual susceptibility, often exhibiting familial clustering [19]. Furthermore, environmental factors and stress can also influence the onset of alopecia areata [22]. Overall, the molecular mechanisms of alopecia areata appear to involve a complex interplay of abnormal immune responses, genetic factors, and environmental and stress-related factors [23]. Accordingly, the aim of this study was to identify candidate molecules through RNA-sequencing that may be associated with hair follicle development in lambs and to explore the underlying mechanisms using in vitro and in vivo models.

In this study, we performed multi-omics analyses using lamb models exhibiting different wool phenotypes, including miRNA sequencing, mRNA sequencing, and proteomic sequencing. We identified *miR-199a-3p* as the sole significantly upregulated small RNA in the skin tissue of fine wool lambs. Further integrated analysis of mRNA and proteomic sequencing revealed that *miR-199a-3p* possesses the potential to regulate hair follicle development via the PTPRF/β-catenin axis. Thus, we established miRNA injection models in sheep veins and mouse skin to validate the regulatory role of *miR-199a-3p* in hair follicle development. Surprisingly, *miR-199a-3p* not only regulates hair follicle development in mice but also in lambs, and these animal models also suggest a potential molecule associated with alopecia areata, which provides potential treatment options.

## 2. Results

### 2.1. Mir-199a-3p Is a Potential Regulator of Wool Development in Fine-Wool Sheep

**Figure 1:**
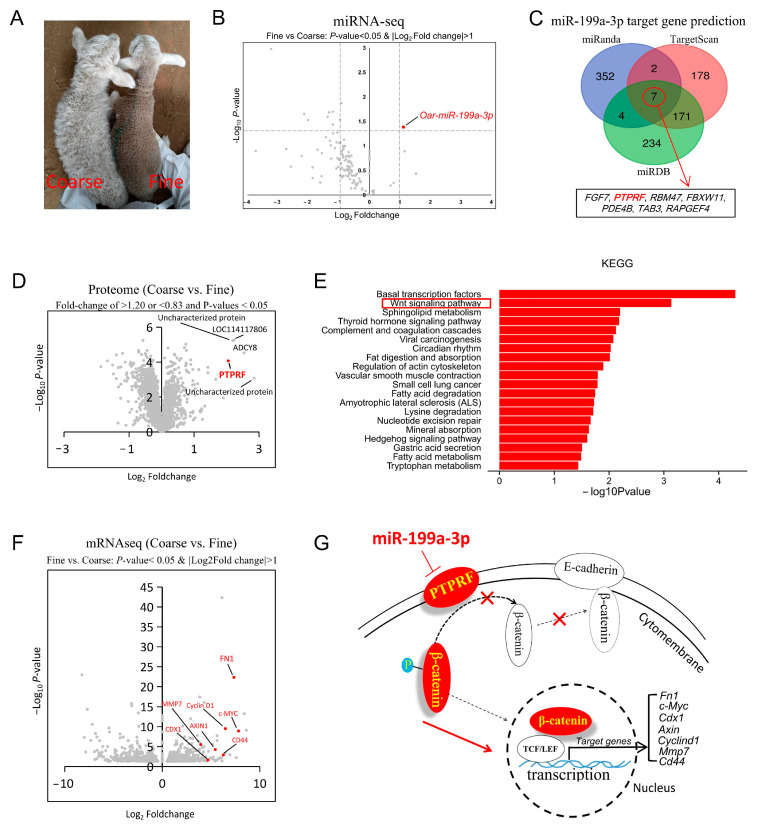
Key miRNAs associated with fine-wool development and their potential signaling pathways. (**A**) Appearance of 1-month-old coarse- and fine-wool lambs. (**B**) Volcano plot of differentially expressed miRNAs, n = 3. (**C**) Venn diagram of *miR-199a-3p* target genes predicted using three databases: miRanda, TargetScan, and miRDB. (**D**) Volcano plot of differentially expressed proteins, n = 3. (**E**) Functional enrichment analysis of the upregulated differentially expressed genes in the fine-wool group using the online tool DAVID (https://david.ncifcrf.gov/tools.jsp. accessed on 8 February 2021). (**F**) Volcano plot of differentially expressed mRNAs, n = 3. (**G**) Candidate signaling pathways involved in the regulation of hair follicle development via *miR-199a-3p* identified in this study.

Skin tissues from 1-month-old coarse- and fine-wool lambs were split into three parts: one for miRNA sequencing, one for proteomics, and one for mRNA sequencing (Figure 1A,B,D,F). The miRNA sequencing results showed that *miR-199a-3p* was the only small RNA significantly overexpressed in the fine-wool group (Figure 1B). Integrated analysis of predicted *miR-199a-3p* target genes and DEPs from proteome screening revealed that PTPRF was the most likely target gene of *miR-199a-3p* (Figure 1C,D and Table S3). The mRNA sequencing results showed that the upregulated DEGs in the fine-wool group were significantly enriched in the Wnt-signaling pathway (Figure 1E). Further, the target genes of β-catenin, a key transcription factor acting downstream of the Wnt-signaling pathway [24], were among the top upregulated DEGs in the fine-wool group, both in terms of multiplier and significance (Figure 1F). Previous studies have indicated that PTPRF regulates the activity of the Wnt-signaling pathway by altering the localization of β-catenin in the cell membrane and nucleus [25,26,27]. Based on these findings, we hypothesized that *miR-199a-3p* controls the development of fine wool by regulating the PTPRF/β-catenin pathway (Figure 1G). Moreover, the overexpression of *miR-199a-3p* was accompanied by the significant suppression of immune system-related marker genes, including the interleukins IL15 and IL13 (Figure 1D,F,G).

### 2.2. MiR-199a-3p Induces Activation of the Wnt Pathway by Targeting PTPRF In Vitro

**Figure 2:**
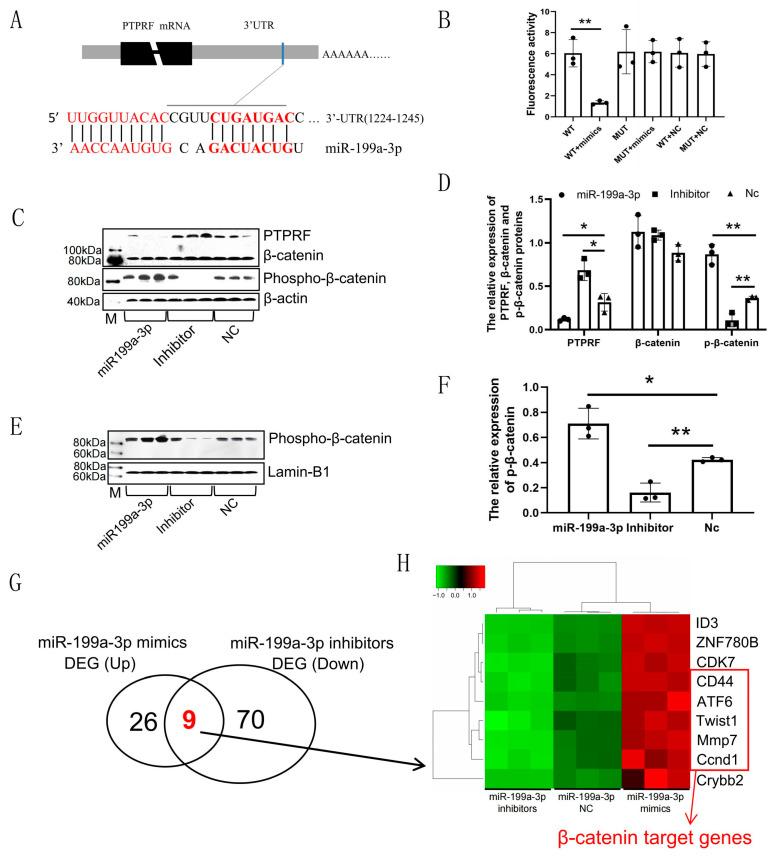
The *miR-199a-3p* target PTPRF activates the Wnt pathway in HEK293T cells. (**A**) Illustration of the predicted *oar-miR-199a-3p* target position. (**B**) Luciferase reporter assay results (** *p* < 0.001, n = 3). (**C**) Expression levels of PTPRF, β-catenin, and phosphorylated β-catenin at Tyr 142 (phosphor-β-catenin) proteins in HEK293T cells after overexpressing *miR-199a-3p* mimics and inhibitors. (**D**) Analysis of the western blot results in C using image J (version number: 1.8.0) (* 0.01 < *p* < 0.05, ** *p* < 0.01, n = 3). (**E**) Expression level of phospho-β-catenin in the HEK293T cell nucleus after the overexpression of *miR-199a-3p* mimics, inhibitors, and negative control. (**F**) Analysis of the western blot results in E using image J (version number: 1.8.0) (* 0.01 < *p* < 0.05, ** *p* < 0.01, n = 3). (**G**) Venn diagram of differentially expressed genes (DEGs) comparing upregulated genes in the *miR-199a-3p* mimics group and downregulated genes in the *miR-199a-3p* inhibitors group. (**H**) Heatmap of mRNA expression levels of co-expressed genes between upregulated genes in the *miR-199a-3p* mimics group and downregulated genes in the *miR-199a-3p* inhibitors group, n = 3.

To validate the regulatory role of *miR-199a-3p* in the PTPRF/β-catenin axis, we constructed mutant and wild-type vectors containing the target sites. These vectors were divided into six combinations and transfected into HEK293T cells as follows: wild-type vector, wild-type vector + *miR-199a-3p* mimic, mutant vector, mutant vector + *miR-199a-3p* mimic, wild-type vector + negative control mimic, and mutant vector + negative control mimic (Figure 2A). The fluorescence activity of the wild-type vector + *miR-199a-3p* mimic group was significantly lower than that of other groups (*p* < 0.001), and there were no significant differences among the other groups (Figure 2B). These results indicated that *miR-199a-3p* could target the 3′-untranslated region (UTR) of the PTPRF gene (Figure S1).

To further explore the molecular mechanism underlying the regulatory role of *miR-199a-3p*, HEK293T cells were divided into three groups: one group was transfected with an *miR-199a-3p* mimic, one group was transfected with an *miR-199a-3p* inhibitor, and the last group was transfected with negative control (Nc) mimics. The cells were collected on Day 6 after transfection. The protein level of PTPRF decreased gradually with increasing days after mimic transfection (Figure 2C,D). Although the total expression of β-catenin remained unchanged, the tyrosine 142 phosphorylated β-catenin (PTyr142 β-catenin) level increased following mimic transfection (Figure 2C,D). The content of PTyr142 β-catenin in the nucleus also increased with increasing time after mimic transfection (Figure 2E,F). The opposite trend was found for HEK293T cells transfected with the *miR-199a-3p* inhibitor (Figure 2C–F). Further, there were no significant changes in the protein levels of PTPRF, β-catenin, and PTyr142 β-catenin in HEK293T cells of the Nc group (Figure 2C–F).

The cell viability assay showed that the activity of HEK293T cells in the mimic group was significantly higher than that in the Nc group (Figure S2A, *p* < 0.001), and the viability of HEK293T cells transfected with the inhibitor was significantly lower than that in the Nc group (Figure S2B, *p* < 0.001). These results indicated that *miR-199a-3p* could promote the entry of PTyr142 β-catenin into the nucleus by targeting PTPRF to ultimately improve the cellular activity of HEK293T cells. Furthermore, a global survey of *miR-199a-3p* regulatory networks by transcriptome sequencing of transfected HEK293T cells showed a small number of significant DEGs when the mimic and inhibitor groups were compared with the Nc group (Figure 2G). Nine overlapping genes were observed between the upregulated DEGs in the mimic group and the downregulated DEGs in the inhibitor group (Figure 2G,H). Further, six of the overlapped genes are known to be direct target genes of β-catenin, including CD44 [28], ATF6 [29], TWIST1 [29], MMP7 [30], and cyclin D1 (CCND1I) [31] (Figure 2H). This result confirmed that *miR-199a-3p* could activate the Wnt/β-catenin signal pathway by targeting PTPRF (Figure 1G).

### 2.3. Mir-199a-3p Intravenous Injection Activates the Wnt Pathway and Promotes Wool Growth in Lambs

**Figure 3:**
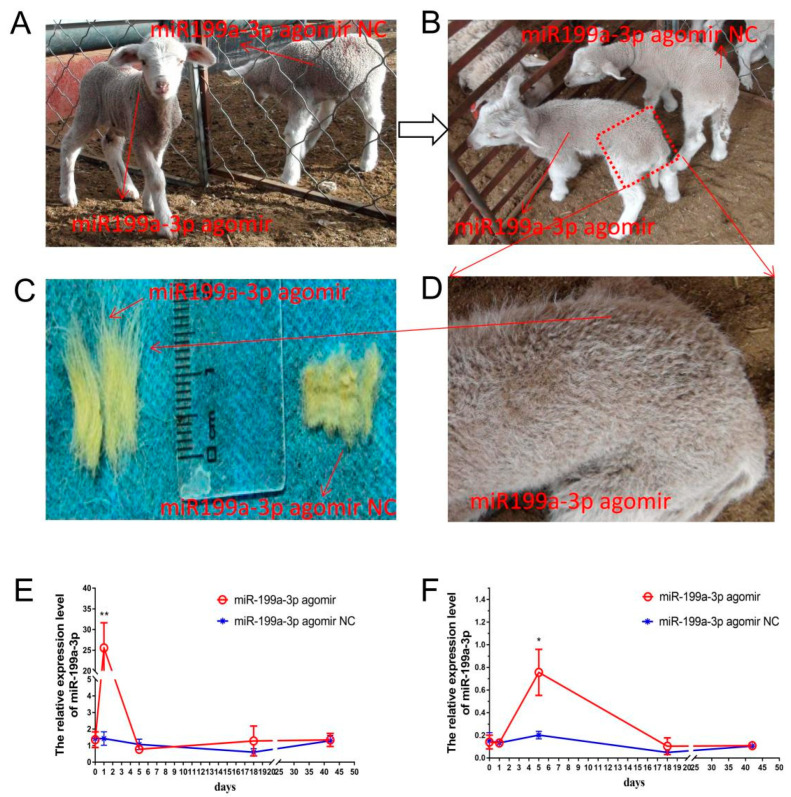
Effects of *miR-199a-3p* agomir jugular injection in lambs. (**A**) Merino lambs used for *miR-199a-3p* agomir jugular injection. (**B**) Eighteen days after *miR-199a-3p* agomir jugular injection. (**C**) Wool fibers between *miR-199a-3p* agomir-injected lambs and non-injected lambs. (**D**) Appearance of *miR-199a-3p*-injected lambs. (**E**) Detection of the *miR-199a-3p* level in lamb blood at different time points after injection (** *p* < 0.01, n = 3). (**F**) Detection of the *miR-199a-3p* level in the lamb skin tissue at different time points after injection (* 0.01 < *p* < 0.05, n = 3). Nc, negative control.

To investigate the precise impact of *miR-199a-3p* and to validate its molecular regulatory mechanism in wool follicle development, we established a live lamb validation model. Eight fine-wool lambs of the same sex (female) were equally divided into two groups. One group received an intravenous injection of an *miR-199a-3p* agomir (IV-agomir), and the other received an intravenous injection of an *miR-199a-3p* agomir negative control (IV-agomir-Nc) (Figure 3A). The level of *miR-199a-3p* in the blood peaked on the first day after injection and gradually returned to the same level as that in the control group within 5 days after injection (Figure 3E). The expression of *miR-199a-3p* in the skin peaked on the fifth day after injection and gradually returned to the same level as that in the control group within 18 days after injection (Figure 3F). At 18 days after injection, the wool length in the IV-agomir group was significantly longer than that in the IV-agomir Nc group (Figure 3B–D and Figure S3).

**Figure 4:**
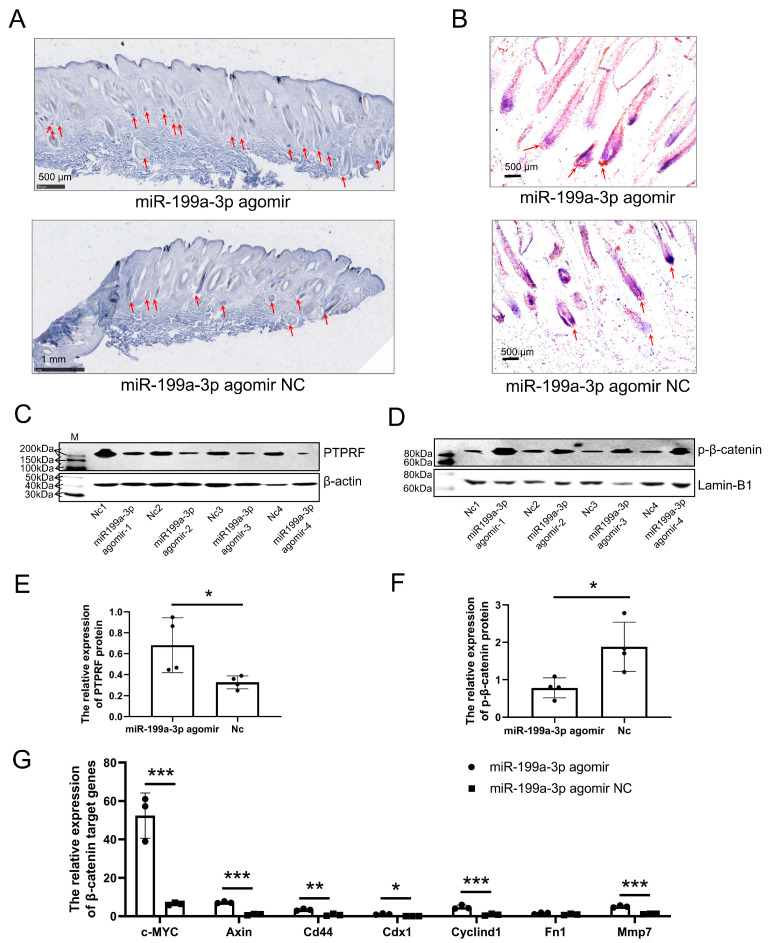
*MiR-199a-3p* targets PTPRF to activate the Wnt pathway in vivo in the lamb model. (**A**) Localization of *miR-199a-3p* expression in the lamb skin was determined using in situ hybridization; red arrows indicate the dermal papilla. The blue color represents positive signals; the deeper the blue, the higher the expression level of miR-199a-3p. (**B**) Localization of phosphorylated β-catenin at Tyr142 (p-β-catenin) protein expression in the lamb skin was determined using immunohistochemistry; red arrows indicate the dermal papillas. the reddish-brown color represents positive signals, and the deeper the color, the higher the expression level of p-β-catenin (**C**) Expression level of the PTPRF protein after intravenous injection with an *miR-199a-3p* agomir and *miR-199a-3p* agomir negative control (Nc). (**D**) Expression level of the p-β-catenin protein after intravenous injection with *miR-199a-3p* agomir and *miR-199a-3p* agomir Nc. (**E**) Analysis of the western blot results in C using Image J (version number: 1.8.0) (* 0.01 < *p* < 0.05, n = 4). (**F**) Analysis of the western blot results in D using Image J (version number: 1.8.0) (* 0.01 < *p* < 0.05, n = 4); (**G**) Quantitative PCR analysis of the mRNA levels of β-catenin target genes after intravenous injection with *miR-199a-3p* agomir and *miR-199a-3p* agomir Nc (* 0.01 < *p* < 0.05, ** *p* < 0.01, *** *p* < 0.001, n = 3).

On the fifth day after the injection, in situ hybridization showed that the expression of *miR-199a-3p* was the most obvious in the dermal papilla cells of the IV-agomir group. These cells are capable of producing fine wool fibers (Figure 4A). Immunohistochemical analyses showed that PTyr142 β-catenin expression was also concentrated in the dermal papilla cells of the IV-agomir group (Figure 4B). These results indicated that *miR-199a-3p* might regulate the growth of wool follicles through the dermal papilla cells. Further, western blot experiments showed the inhibition of the PTPRF protein expression in the lamb skin and an increase in the nuclear level of the PTyr142 β-catenin protein in the IV-agomir group (Figure 4C–F). The qPCR assay showed the activation of β-catenin target genes in the skin of the IV-agomir group (Figure 4G). These results demonstrated that *miR-199a-3p* could promote fine wool growth by activating the Wnt/β-catenin-signaling pathway.

### 2.4. Establishment of a Mouse Model of Alopecia Areata through Local Intradermal Injection of miR-199a-3p Antagomir

**Figure 5:**
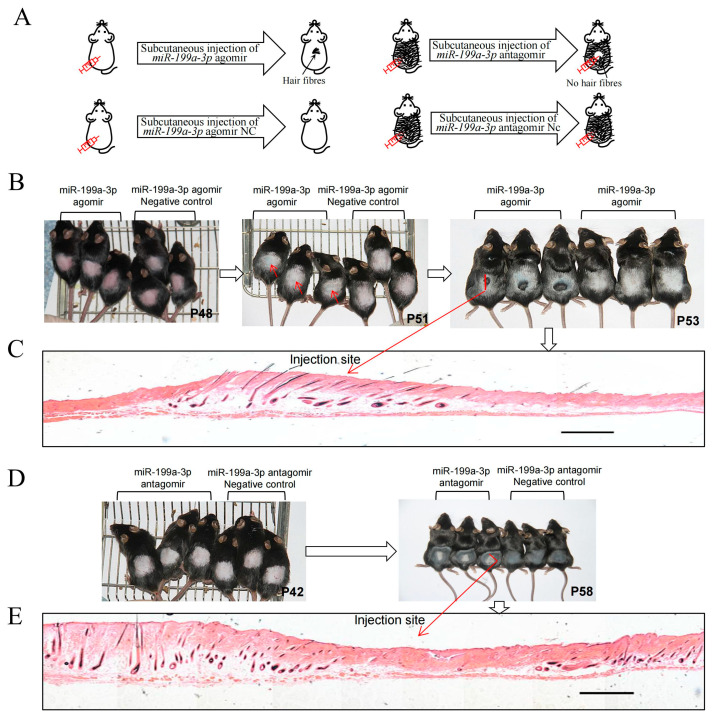
Establishment of the alopecia areata mouse model via intracutaneous injection of *miR-199a-3p*. (**A**) Diagram of the *miR-199a-3p* antagomir-intracutaneous-injected mouse model (left), diagram of the *miR-199a-3p* agomir-intracutaneous-injected mouse model (right). (**B**) Intracutaneous injection of *miR-199a-3p* antagomir and *miR-199a-3p* antagomir negative control in mice. Red arrows indicate injection sites. (**C**) Hematoxylin–eosin staining of the *miR-199a-3p* antagomir injection site. The bar: 1.5 mm (**D**) Intracutaneous injection of *miR-199a-3p* agomir and *miR-199a-3p* agomir negative control in mice. (**E**) Hematoxylin–eosin staining of the *miR-199a-3p* agomir injection site. The bar: 1.5 mm.

To validate the hypothesis regarding the involvement of *miR-199a-3p* in mediating the regulation of the PTPRF/β-catenin axis in alopecia areata, we established a novel mouse model of alopecia areata. Six 48-day-old mice (with telogen hair follicles) of the same sex (female) were selected from two litters. Three mice were injected intracutaneously with an *miR-199a-3p* agomir (IC-agomir), and the other three were intracutaneously injected with an *miR-199a-3p* agomir negative control (IC-agomir-Nc) (Figure 5A). On the fourth day (at 51 days of age) after injection, the skin color of the IC-agomir group was significantly darker than that of the IC-agomir-Nc group (Figure 5B, middle). On the sixth day (53 days old), the injection site in the IC-agomir group turned black and showed hair growth (Figure 5B, right). No new hair was found in the IC-agomir Nc group and at the non-injection site (Figure 5B, right, Figure 5C). Moreover, the IC-agomir group showed anagen entry 5 days earlier than the wild-type group, and their skin color turned completely black (58 days old).

Further, six 42-day-old mice (with catagen hair follicles) of the same sex (female) were divided into two equal groups intracutaneously injected with *miR-199a-3p* antagomir (IC-antagomir) and *miR-199a-3p* antagomir control (IC-antagomir-Nc), respectively (Figure 5A). From the third day of continuous injection (44 days of age), the skin color on the backs of mice showed obvious changes. After 14 days (58 days of age), the skin at the injection site in the IC-antagomir group was gray, whereas that at the non-injection site and in the IC-antagomir Nc group was black, similar to that in the 58-day-old wild-type mice showing black skin color in the anagen phase (Figure 5D, right and Figure 5E).

### 2.5. Mir-199a-3p Induces Comprehensive Changes in Immune-Related Genes at the Intradermal Injection Site through Regulation of the Wnt Signaling Pathway

**Figure 6:**
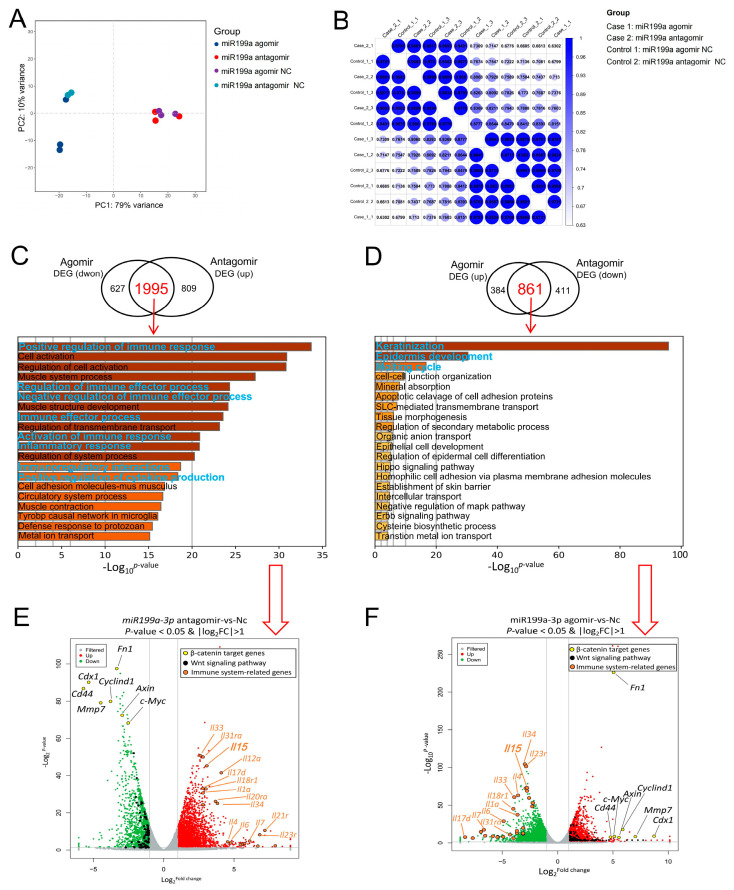
Skin transcriptome analysis in the *miR-199a-3p*-injected mouse model. (**A**) Principal component analysis of the gene expression levels among intracutaneous injection groups, n = 3. (**B**) Correlation analysis of transcriptome profiles among the samples. (**C**) Venn diagram between downregulated differentially expressed genes (DEGs) in the *miR-199a-3p* agomir group and upregulated DEGs in the *miR-199a-3p* antagomir group with functional enrichment analysis of the overlapped DEGs indicated by the arrow (Metascape online tool; https://metascape.org/gp/index.html). Accessed on 20 February 2021 (**D**) Venn diagram between upregulated DEGs in the *miR-199a-3p* agomir group and downregulated DEGs in the *miR-199a-3p* antagomir group, with functional enrichment analysis of the overlapped DEGs indicated by the arrow (https://metascape.org/gp/index.html). Accessed on 20 February 2021 (**E**) Volcano plots of DEGs between the *miR-199a-3p* agomir and negative control (Nc) groups, n = 3. The light gray dots represent genes with non-significant differences. Solid gray lines indicate differential expression significance thresholds. (**F**) Volcano plots of DEGs between the *miR-199a-3p* antagomir and Nc groups, n = 3. The light gray dots represent genes with non-significant differences. Solid gray lines indicate differential expression significance thresholds.

To verify if the established model of alopecia areata corresponds to the pathogenic mechanism of the disease, we collected skin tissues from the injection and non-injection sites of the IC-agomir, IC-agomir-Nc, IC-antagomir, and IC-antagomir-Nc groups for transcriptome sequencing. Principal component analysis showed that the gene profiles of the experimental and control groups were completely separated along PC1 (Figure 6A). Correlation analysis also showed significant differences in expression levels between groups (Figure 6B). Notably, there was a large overlap between the downregulated DEGs in the IC-agomir group and the upregulated DEGs in the IC-antagomir group, accounting for 76.1% and 71.1% of their total downregulated and upregulated DEGs, respectively (Figure 6C). This overlapping gene set was enriched in many signaling pathways known to be related to the immune system. This suggests that the mouse immune system as a whole was boosted when *miR-199a-3p* antagomir was overexpressed.

There was also a large overlap between the upregulated DEGs in the IC-agomir group and the downregulated DEGs in the IC-antagomir group, accounting for 69.2% and 67.7% of the total upregulated and downregulated DEGs, respectively (Figure 6D). This overlapping gene set was enriched in many signaling pathways related to hair follicle development, including keratinization, keratinocyte differentiation, and the molting cycle (Figure 6D). Furthermore, the Wnt-signaling pathway emerged as the top-ranking candidate in the protein–protein interaction analysis (Figure S4). The transcriptomic data showed that the target genes of β-catenin were upregulated in the IC-agomir group (Figure 6F) and downregulated in the IC-antagomir group (Figure 6E) compared with those in the control group and were within the top-ranking DEGs in terms of both fold-change and significance (Figure 6E,F). In addition, reported alopecia areata-related genes were upregulated in the IC-antagomir group (Figure 6E) and downregulated in the IC-agomir group (Figure 6F).

### 2.6. Mir-199a-3p Regulates the Characteristics of Alopecia Areata through the PTPRF/Phospho-β-Catenin (Tyr142) Axis

**Figure 7:**
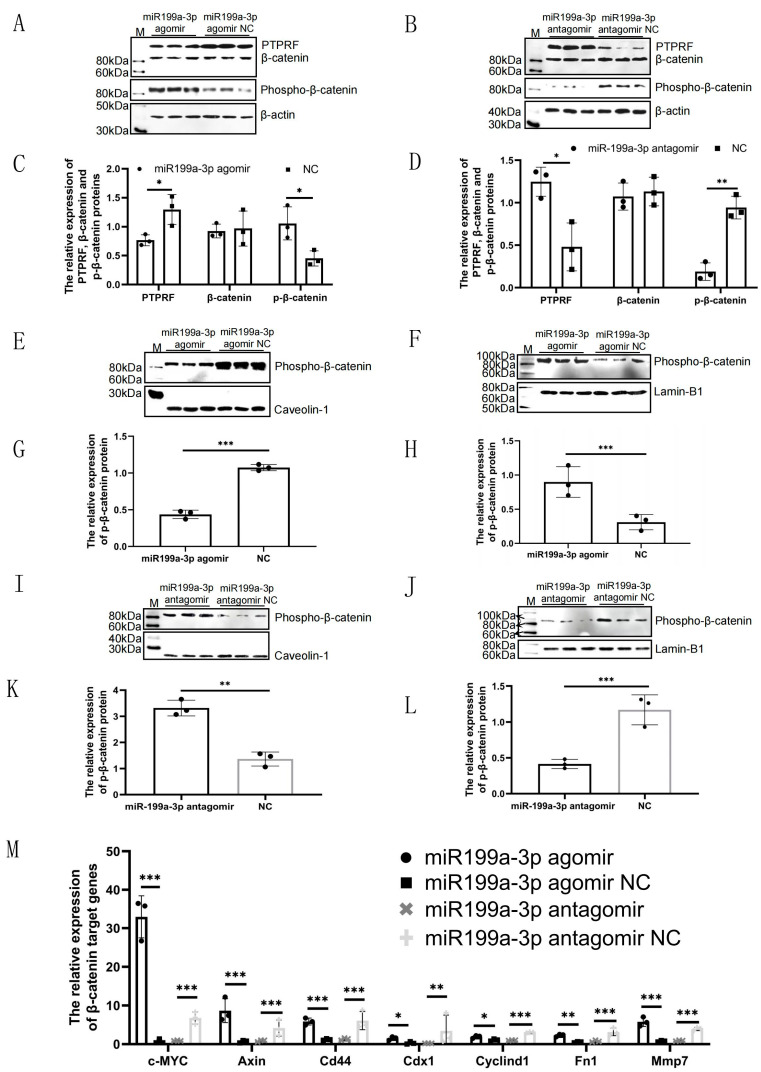
*MiR-199a-3p* targets PTPRF to increase the phospho-β-catenin (Tyr142) level in the cell nuclei of mice. (**A**) Expression levels of PTPRF, β-catenin, and phosphor-β-catenin proteins at the intracutaneous injection site of the *miR-199a-3p* agomir and *miR-199a-3p* agomir negative control groups. (**B**) Expression levels of PTPRF, β-catenin, and phosphor-β-catenin proteins at the intracutaneous injection site in the *miR-199a-3p* antagomir and *miR-199a-3p* antagomir negative control (Nc) groups. (**C**) Analysis of the western blot results in (**A**) using Image J (version number: 1.8.0) (* 0.01 < *p* < 0.05, n = 3). (**D**) Analysis of the western blot results in (**B**) using Image J (version number: 1.8.0) (* 0.01 < *p* < 0.05, ** *p* < 0.01, n = 3). (**E**) Expression level of phosphor-β-catenin in the cell membrane at the intracutaneous injection site in the *miR-199a-3p* agomir and *miR-199a-3p* agomir Nc groups. (**F**) Expression level of phosphor-β-catenin in the cell nucleus at the intracutaneous injection site in the *miR-199a-3p* agomir and *miR-199a-3p* agomir Nc groups. (**G**) Analysis of the western blot results in E using Image J (version number: 1.8.0) (*** *p* < 0.001, n = 3). (**H**) Analysis of the western blot results in F using Image J (version number: 1.8.0) (*** *p* < 0.001, n = 3). (**I**) Expression level of phosphor-β-catenin in the cell membrane at the intracutaneous injection site in the *miR-199a-3p* antagomir and *miR-199a-3p* antagomir Nc groups. (**J**) Expression level of phosphor-β-catenin in the cell nucleus at the intracutaneous injection site in the *miR-199a-3p* antagomir and *miR-199a-3p* antagomir Nc groups. (**K**) Analysis of the western blot results in I using Image J (version number: 1.8.0) (** *p* < 0.01, n = 3). (**L**) Analysis of the western blot results in J using Image J (version number: 1.8.0) (*** *p* < 0.001, n = 3). (**M**) Relative expression of β-catenin target genes in the mouse skin at the intracutaneous injection site (* 0.01 < *p* < 0.05, ** *p* < 0.01, *** *p* < 0.001, n = 3).

The findings above suggested that the successful establishment of the mouse model of alopecia areata was achieved through the *miR-199a-3p*-mediated activation of the Wnt-signaling pathway. A series of molecular experiments were then conducted to validate this hypothesis. qPCR verification showed that the mRNA levels of β-catenin target genes were significantly increased in the IC-agomir group and significantly decreased in the IC-antagomir group compared with those of controls (Figure 7M). Western blot experiments showed that *miR-199a-3p* inhibited the protein expression of PTPRF in the IC-agomir group, resulting in increased entry of PTyr142 β-catenin into the nucleus (Figure 7A,C,E–H). In the IC-antagomir group, the inhibitory effect of *miR-199a-3p* on the protein expression of PTPRF decreased, which also resulted in the decreased entry of PTyr142 β-catenin into the nucleus (Figure 7B,D,I–L). These findings further indicated that *miR-199a-3p* was necessary for Wnt/β-catenin activation and consequently promoted the development of hair follicles in vivo.

## 3. Discussion

In this study, we showed that PTyr142 β-catenin transcription activity could be triggered by the newly identified interaction between *miR-199a-3p* and PTPRF using lamb and mouse models in vivo, as well as in vitro functional studies in a human cell line. At the cellular level, the elevated expression of *miR-199a-3p* inhibits the translation of its target gene *PTPRF*, subsequently causing the translocation of β-catenin into the cell nucleus and activation of the Wnt-signaling pathway. Conversely, when the expression of *miR-199a-3p* is suppressed, the activity of the Wnt-signaling pathway is inhibited. At the in vivo level in lambs and mice, we demonstrate that *miR-199a-3p* regulates wool and mouse hair development by targeting the PTPRF/β-catenin axis. Furthermore, in vivo experiments in mice resulted in differential expression of a series of alopecia-related marker genes, including several immune response-signaling genes (Figure 1F and Figure 6E,F). Aberrant immune responses are considered a major cause of alopecia areata, and interleukin-15 is considered a key factor in the development of the condition [32,33]. Therefore, we propose a hypothesis that *miR-199a-3p* may exhibit a similar regulatory mechanism in wool follicle development as observed in the pathogenesis of alopecia areata.

β-catenin phosphorylation at Tyr142 is reported to facilitate the release of β-catenin from the cell membrane and its translocation to the nucleus [25]. Further, when tyrosine kinase (c-Met) is activated by hepatocyte growth factor/serum ferritin [26], the β-catenin/E-cadherin complex is disintegrated, and β-catenin enters the cytoplasm from the cell membrane; β-catenin then enters the nucleus with the help of BCL9-2 (a protein homologous to Drosophila Legless) [34]. However, mutant β-catenin phosphorylated at Tyr142 cannot bind to BCL9-2 [35] and loses its ability to enter the nucleus, resulting in significantly decreased transcriptional activity [26]. Similar studies have shown that when c-Met is mutated, β-catenin phosphorylated at Tyr142 accumulates in the cell, which increases β-catenin transcriptional activity and the expression levels of the target genes of β-catenin/T-cell factor (a Wnt transcription factor) [27]. Here, we provide solid evidence that *miR-199a-3p* plays an important role in regulating hair follicle development by inducing PTyr142 β-catenin nuclear translocation.

As an important component of the Wnt-signaling pathway, β-catenin plays a critical role in hair follicle cycle development and hair follicle stem cell differentiation [36]. The activation of β-catenin at the background level leads to the beginning of hair follicle morphogenesis in the adult skin, which is otherwise a specific phenomenon at the embryonic stage [37]. Conversely, the specific inactivation of β-catenin transforms stem cells into epithelial cells rather than hair follicle cells [38]. In β-catenin-overexpressing transgenic mice with amino-terminal truncation, the proliferation of hair follicle bulge region cells, volume of hair follicles, and cyclin-D expression were increased, whereas the number of labeled cells was decreased [39].

In recent years, research has shown that the aberrant activation of the Wnt-signaling pathway is associated with the development and progression of alopecia areata [40,41]. Studies have found that the activity of the Wnt-signaling pathway is reduced in the hair follicles of patients with alopecia areata, causing disruption of the hair follicle growth cycle and ultimately resulting in hair loss [42]. Similar results were obtained in our study with animal models, where the local intradermal injection of *miR-199a-3p* antagomir led to a decrease in the Wnt signaling-pathway activity and the inhibition of hair follicle development at the injection site. Furthermore, the expression of β-catenin, as an indispensable component of the downstream transcription factor complex in the Wnt-signaling pathway, is significantly downregulated in the hair follicles of patients with alopecia areata [43]. This downregulation leads to aberrant Wnt-signaling pathway activity, disrupting normal hair follicle development and hair growth cycle, ultimately resulting in hair loss [44]. Further research suggested that the low expression of the β-catenin gene may be closely associated with immune system abnormalities and alterations in the function of hair follicle stem cells in patients with alopecia areata [45]. In the present study, using both lamb and mouse models, the PTPRF/β-catenin axis was identified as the primary target pathway of *miR-199a-3p*. The inhibition of β-catenin transcriptional activity mediated by *miR-199a-3p* indeed exhibited alopecia features, including abnormal activation of the local immune system and restricted hair growth. Therefore, an in-depth investigation of the role of the β-catenin gene in alopecia areata can contribute to gaining a better understanding of the mechanistic basis of the disease and provide potential avenues for the development of novel therapeutic strategies.

## 4. Materials and Methods

### 4.1. Animal and Sample Collection

C57/BL mice in this study were obtained from the Shanghai Research Center for Model Organisms (Shanghai, China) and 6 female mice from two litters were selected for intracutaneous injection experiment. Eight Chinese Aohan Merino lambs (female) were used for intravenous injection. The skin tissues of mice and lambs were collected and divided into two parts. One was used for HE staining, immunohistochemistry, and in situ hybridization (ISH), and the other was placed in liquid nitrogen for subsequent RNA-seq, Q-pcr, and Western blot. The lamb blood was collected for the follow-up Q-pcr of *miR-199a-3p*.

### 4.2. Cell Culture and Histological Analysis

HEK293T cells were obtained from the Institute of Biochemistry and Cell Biology, Chinese Academy of Science, P. R. China. All cells were cultured in DMEM, plus 10% FBS and penicillin/streptomycin. Cells were maintained at 37 °C with 5% CO_2_. All reagents used for cell culture were purchased from Invitrogen/Gibco (Carlsbad, CA, USA). Skin samples from back of lambs and mice were embedded in O.C.T. tissue freezing medium (CM1900; Leica, Nussloch, Germany) and sectioned (9 μm thickness). Sections were stained with hematoxylin and eosin (H&E) (C0105S, Beyotime, Beijing, China).

### 4.3. Dual Luciferase Assay

HEK293T cells were used to validate *miR-199a-3p* target. Cells were seeded into 24-cell plates (Corning Incorporated, New York, NY, USA) and transfected 24 h later with Lipofectamine 2000 (L3287-1ML, Sigma–Aldrich, St. Louis, MO, USA). The *miR-199a-3p* was co-transfected with 150 ng of psicheck2-PTPRF-fragment and 50 pmol of *miR-199a-3p* mimics, or negative control mimics (Ribobio, Guangzhou, China). The fragments in psicheck2-PTPRF-fragment were designed and synthesized by Shanghai Generay Biotech Co., Ltd. (Generay Biotech Co, Shanghai, China) and constructed into the psiCheck2 vector. Forty-eight hours after transfection, firefly and renlilla luciferase activities were measured using the dual-luciferase reporter kit (E1910, Promega, Madison, WI, USA). Assays were repeated three times.

### 4.4. The Detection of Cell Viability

Cell viability was detected by CellTiter–Lumi assay Plus kit (C0068S, Beyotime, Beijing, China). The specific steps are as follows: (A) Using a 96-well plate detected by chemiluminescence detection; (B) Inoculating 100 μL cells in each well, and ensuring that the number of cells in each well is less than 100,000, and setting a culture medium hole without cells as a negative control. Cells were cultured according to the conventional method of the cell culture; (C) Preparation of detection reagent (thawing and freezing at room temperature, CellTiter–Lumi Plus luminescence detection reagent (C0065, Beyotime, China) was thawed at room temperature. According to the amount of 100 μL per well in 96-well plates, appropriate amount of CellTiter–Lumi fluorescence Plus, photoluminescence detection reagent was chosen and balanced to room temperature). (D) Cell viability test: withdraw the cell culture plate and balance at room temperature for 10 min. Then, 100 μL CellTiter–Lumi Plus photoluminescence detection reagent was added to each well of 96-well plate. Shake at room temperature for 2 min and incubate at room temperature (about 25 °C) for 10 min. Chemiluminescence detection (Infinite 200 Pro, Tecan, Switzerland) was performed by using a multi-function enzyme labeling instrument. The relative viability of the cells was calculated directly from the chemiluminescence readings.

### 4.5. RNA Extraction and Q-PCR

Total RNA was extracted from HEK293Tcells, lambs, and mice skin tissue using TRIzol reagent according to the manufacturer’s instructions (15596026, Invitrogen, Carlsbad, CA, USA). cDNA synthesis was performed with 1 μg of total RNA, following the protocol accompanying the FastQuant RT Kit (KR116, TIANGEN, Beijing, China). To detect the relative expression of c-MYC, Axsin, Cd44, Cdx1, Cyclind1, Fn1, and Mmp7 mRNA, real-time PCR was performed with the primers shown in Table S1, using β-actin as the reference gene. Primers for c-MYC, Axsin, Cd44, Cdx1, Cyclind1, Fn1, Mmp7, and β-actin were synthesized by Sangon (Sangon, Shanghai, China). The reverse transcription of *miR-199a-3p* and *U6* was conducted according to the experimental procedure of kit TaqManTM MicroRNA Revverse Transcription Kit (#4366596, Thermo Fisher Scientific, Waltham, MA, USA). Among them, every 15 μL of reverse transcription reaction system needs 10 ng of total RNA, and the reverse transcription reaction system includes 7 μL of Mastermix mixture (see kit instructions), 3 μL of 5 × RTprimer, and 5 μL of RNA samples. Q-pcr selected SYBR Green qPCR mix kit and was conducted in BioRadCF × 96 (BioRad, Hercules, CA, USA) quantitative instrument. Reaction procedure: pre-denatured (30 s) at 95 °C, denaturing (10 s) at 95 °C, annealing (30 s) at 60 °C, extension (30 s) at 72 °C, for 40 cycles. The repeats of three holes were set for each sample, and U6 was used as the internal reference. The relative expression between groups was calculated by 2^−ΔΔCT^ [46]. SAS9.1 was used for statistical analysis, while *p* < 0.05 was selected as the level of significance.

### 4.6. Total Protein Extraction

Total protein was extracted from the skin tissue of lambs, mice, and HEK293T cells with ice-cold RIPA lysis buffer containing PMSF (phenylmethylsulfonyl fluoride) (P0013B, Beyotimes, Beijing, China). Samples were centrifuged at 4 °C for 30 min at 12,000× *g*. Total protein concentration was measured using a Braford protein assay kit (PA102, Tiangen, Beijing, China).

### 4.7. Cell Membrane Protein Extraction

Cell membrane proteins were completed according to Membrane and Cytosol Protein Extraction Kit (P0033, Beyotime, Beijing, China). The extraction of cell membrane protein was divided into tissue and cell. HEK293T cell membrane protein extraction should first cultivate about 2000-50-illion cells, wash them with PBS, scrape off the cells with cell scrapers, and blow down the cells with a pipette. Centrifuge to collect cells, absorb the supernatant, leaving cells to precipitate, and set aside. A small number of cells were counted, and the remaining cells were precipitated by centrifugation at 4 °C and 600× *g* for 5 min. The supernatant was discarded, followed by centrifugation at 4 °C and 600× *g* for 1 min to precipitate the residual liquid on the wall of the centrifuge tube and further precipitate the cells, and there is an attempt to absorb the residual liquid. Add 1 mL of membrane protein extraction Reagent A with PMSF before using 2000-50-million cells, gently and fully suspend the cells, and place them in an ice bath for 10–15 min. To extract the membrane protein of skin tissue, extract roughly 100 mg of tissue and cut it into small tissue fragments as carefully as possible with scissors. Add 1 mL of PMSF membrane protein extraction Reagent A before use, gently suspend the tissue fragments, and place in the ice bath for 10–15 min. Transfer the cell suspension or tissue sample to an ice bath precooled glass homogenizer of appropriate size, homogenizing for about 30–50 times. About 2–3 microliters of cells or tissue homogenate were dropped on the cover slide and observed under the microscope. If 70–80% of the cells had no perinuclear halo (a shiny ring around the nuclei) and intact cell morphology, indicating that the cells had been fully broken, the next experiment would be conducted. Centrifuge at 4 °C, 700× *g* for 10 min, and carefully collect the supernatant into a new centrifuge tube. Do not touch the precipitate when absorbing the supernatant. The cell membrane fragments were precipitated by centrifugation at 4 °C and 14,000× *g* for 30 min. Try your best to suck up the supernatant and gently touch the precipitation, or even absorb a very small amount of precipitation. Add membrane protein extraction reagent B200 microliter (if necessary, can also be increased to 300 microliters), the highest speed of violent Vortex5 s re-suspension precipitation, in ice bath for 5–10 min. Repeat the previous steps of vortex and ice bath incubation 1–2 times to fully extract membrane proteins. Then, it wass centrifuged at 4 °C and 14,000× *g* for 5 min, and the supernatant was collected as cell membrane protein solution.

### 4.8. Nuclear Protein Extraction and Western Blot

Nuclear proteins were completed according to Nuclear and Cytoplasmic Protein Extraction Kit (P0033, Beyotime, Beijing, China). For HEK293T cells, wash them with PBS, scrape off the cells with a cell scraper, and blow them down with a pipette. Centrifuge to collect cells and attempt to absorb the supernatant, leaving cells to precipitate and set aside. (A) Two hundred microliters of cytoplasmic protein extraction reagent added with PMSF was added every 20 microliters of cell precipitation. (B) The highest speed and violent Vortex 5 s, the cell precipitation was completely suspended and dispersed. (C) Ice bath for 10–15 min. (D) Add cytoplasmic protein extraction reagent B10 microliter. (E) The highest-speed violent Vortex 5 s, ice bath 1 min. (F) The highest-speed and violent Vortex 5 s, centrifuge at 4 °C 12,000–16,000× *g* for 5 min. (G) For precipitation, completely absorb the residual supernatant and add 50 microliters of nuclear protein extraction reagent added with PMSF. (H) The highest speed and violent Vortex 15–30 s, the cell precipitation was completely suspended and dispersed. (I) It was then placed again in the ice bath every 1–2 min before high-speed violent Vortex 15–30 s, a total of 30 min. (J), 4 °C 12,000–16,000× *g* centrifugation for 10 min. (K) Immediately absorb the supernatant into a precooled plastic tube, that is, the extracted nuclear protein. For skin tissue samples, cut the tissue into pieces as small as possible. Mix the equivalent cytoplasmic protein extraction reagents A and B according to the proportion of 20:1. The tissue homogenate was prepared by adding PMSF to the final concentration of 1 mM. The tissue and tissue homogenate were mixed according to the proportion of 200 microliters of tissue homogenate per 60 mg and fully homogenized in the glass homogenizer. Homogenization should be conducted in an ice bath, or at 4 °C. After homogenizing, transfer the homogenate to a plastic centrifuge tube and place in an ice bath for 15 min. Centrifuge at 4 °C 1500× *g* for 5 min. In the next step, the nuclear protein of skin tissue can be obtained according to A–K.

Thirty micrograms of total protein, 10 μg of membrane protein, and 10 μg of nucleo protein were separated by SDS–PAGE and transferred to PVDF membranes (Millipore, Billerica, MA, USA). The membranes were probed with specific primary antibodies (sc-135969, anti-PTPRF, Santa Cruz, Hercules, CA, USA) anti-β-catenin (E247, Abcam, Boston Metro, MA, USA) anti-p-β-catenin, pTyr142 (bs-2063R, Bioss, Beijing, China), β-actin (AF2811, Beyotime, Beijing, China), Caveolin-1 (AF0087, Beyotime, Beijing, China), and Lamin B1 (AF1408, Beyotime, Beijing, China), as well as appropriate secondary antibodies anti-rabbit-HRP (A0208, Beyotime, Beijing China) and anti-mouse-HRP (A0192, Beyotime, Beijing China). The PTPRF, β-catenin, p-β-catenin(pTyr142), β-actin, Caveolin-1, and Lamin B1 primary antibodies were diluted to 1:2000, 1:2000, 1:1000, 1:3000, 1:2000, and 1:2000 before use, respectively. The secondary antibodies were diluted to 1:3000.

The full length uncropped original western blots used in their manuscript had been submitted in the Supplementary Files (Figure S5).

### 4.9. RNA-Seq

A total of 4 μg of RNA from each skin and cell sample were used as input material for RNA sample preparation. Ribosomal RNA was removed using the Epicentre Ribo-zero™ rRNA Removal Kit (Epicentre, Madison, WI, USA); the mRNA sequencing libraries were constructed using the NEBNext^®^ Ultra™Directional RNA Library Prep Kit for Illumina^®^ (NEB, Ipswich, MA, USA), according to the manufacturer’s instructions. The index codes were used to attribute the sequences to each sample. Clustering of the index-coded samples was performed on the cBot Cluster Generation system using the TruSeq PE Cluster Kit v3-cBot-HS (Illumina). Subsequently, the mRNA libraries were sequenced on the Illumina Hiseq 2000 platform. The clean reads were obtained from raw reads by removing poly-N regions, reads containing adapters, and low-quality reads (Trimmomatic) [47]. The Q20, Q30, and GC contents of the clean reads were calculated. The human reference genome and annotation files were obtained from the human genome website (Table S2). The mouse reference genome and annotation files were obtained from the mouse genome website (Table S1). The reference genome index was constructed using Bowtie2, and clean paired-end reads were aligned to the reference genome using TopHat [48]. The mapped reads of each sample were assembled using both Cufflinks and Scripture (beta2) with a reference-based approach [49]. The transcripts were either predicted based on the coding potential by the Coding-Non-Coding-Index (CNCI) [50], Coding Potential Calculator, 0.9-r2 (CPC) [51], or Pfam Scan [52], and the phylogenetic codon substitution frequency (PhyloCSF) [53] and all four programs were filtered out. Subsequently, those without coding potential were designated as the candidate mRNA set. We used the PhyloFit [54] program to compute phylogenetic models for conserved and non-conserved regions among species. The phastCons program was used in conjunction with the model and HMM transition parameters to compute a set of conservation scores for the mRNAs. The Cuffdiff algorithm was used to calculate the fragments per kilobase of exon per million fragments mapped (FPKMs) of mRNAs in each sample. The DESeq2 package was employed for the analysis of differential gene expression. Using a model based on the negative binomial distribution, the DEGs with an adjusted *p*-value (*p*-adjust < 0.05; Benjamini–Hochberg multiple test correction).

### 4.10. miRNA-Seq

Total RNA, including small RNA, was extracted by TRIZOL, and the quality of the extracted RNA was tested by NanoDrop2000. RNA integrity was identified by 1.5% agarose gel electrophoresis. One microgram of total RNA was obtained from each sample, and the TruSeq Small RNA Sample Prep Kits (Illumina, San Diego, CA, USA) was used to build a small RNA library. Then, Illumina Hiseq2000 was used for subsequent sequencing, as well as the Ovis genome website (Table S1). The raw data was presented in FASTQ format by removing the adaptors. We selected clean read lengths greater than 18 nt as small RNAs. The DEGs with an *p*-value (*p*-adjust < 0.05; Benjamini–Hochberg multiple test correction) between coarse and fine wool lambs were identified.

### 4.11. Proteome

The lysis buffer (200 μL, it contains 4% SDS, 100 mM DTT, 150 mM Tris-HCl pH 8.0) was used in the suspended skin tissues. Quantification of lysate supernatant by BCA Protein Assay Kit (Bio-Rad, Hercules, CA, USA). Digestion of protein (200 μg for each sample) was performed according to the FASP procedure. The peptide concentration was determined with OD280 by Nanodrop device. Two hundred micrograms of each sample were obtained for protein digestion by the FASP procedure, and the peptide concentration was determined using the Nanodrop device. Peptides were labeled with TMT reagents according to the manufacturer’s instructions (Thermo Fisher Scientific, Waltham, MA, USA), and each aliquot was reacted with one tube of TMT reagent, respectively. Full MS resolutions were set to 120,000 at *m*/*z* 200, and the full MS AGC target was 300% with an injection time of 25 ms. Mass range was set to 350–1400. The raw data were processed in Proteome Discoverer 2.4 (Thermo Fisher Scientific, Waltham, MA, USA) and imported into MaxQuant software (version 1.6.0.16) for data interpretation and according to the database Uniprot_ OvisAries _23084_ UP000002356, the original data were searched (Table S2).

Analysis of the proteome data was performed using Perseus software (version 1.6.1.3), Microsoft Excel (version 2021), and R statistical computing software (version 3.6.3). Differentially expressed proteins were screened using a ratio fold change of >1.20 or <0.83 with a *p* value < 0.05 as the threshold. Expression data were grouped together according to protein level by hierarchical clustering. To annotate the sequences, information was extracted from UniProtKB. For functional enrichment analysis of DEPs, GO and signaling pathway analyses were performed using the online tool David (https://david.ncifcrf.gov/, accessed on 8 February 2021) [55,56] and Metascape (https://metascape.org/gp/index.html, accessed on 20 February 2021) [57], using Fisher’s exact test and also FDR correction for multiple testing. Enriched GO and signaling pathways were nominally statistically significant at the *p* < 0.05 level.

### 4.12. Immunohistochemical

Lamb skin tissues were fixed in 4% paraformaldehyde formalin in PBS at 4 °C overnight, embedded in paraffin, and sectioned at 6 μm. The following antibodies were used for immunostaining: anti-p-β-catenin, pTyr142, bs-2063R, Bioss, China, and HRP-labeled goat anti-rabbit IgG(H + L) (A0208, Beyotime, Beijing, China). The signal was detected using the DAB Horseradish Peroxidase Color Development Kit (P0202, Beyotime, Beijing, China), and the sections were stained with hematoxylin. Photographs were recorded using a microscope (RVL-100-G, ECHO, San Diego, CA, USA).

### 4.13. In Situ Hybridization

In situ hybridization lamb skin tissue samples were divided into three groups according to the type of miRNA probe (EXQON, Vedbaek, Denmark): *miR-199a-3p* group, scramble-miR group, and U6 group. The specific steps are as follows: Prepare longitudinal paraffin sections of skin tissue, the thickness of which requires 5 μm. Dry the slices, put them in a dry-baked dish, and bake them at 60 °C for 45 min. Then, soak the slices in Xylene I, II, and III, respectively, each time for 5 min. Soak in 100% ethanol twice, each time for 5 min. Soak 5 min in 70% ethanol, and soak in 50% alcohol for 5 min. This is the process of deproteinization (DPBS soaking twice, each time for 5 min, and treated with 10 μg/mL protease K at 37 °C every 5 min). Configure the protease buffer in advance and incubate it at 37 °C. Soak in 0.2% glycine (0.2 g glycine + 100 mL DPBS) for 30 s. Soak in DPBS twice for 30 s each time. Then, fix 4% paraformaldehyde for 10 min at room temperature. Soak DPBS twice, each time for 5 min. Pre-hybridization (putting the slice in a wet box, encircling the tissue in the section with a hydrophobic pen and dripping hybrid buffer; the composition of buffer includes 50% formamide 5 mL, 5 × SSC 2.5 mL, 0.1% Tweenly20 0.01 mL, 9.2 mM citric acid, 50 μg/mL heparin 10 μL, 500 μg/mL Yeast RNA 250 μL, DEPC water 1.230 mL). Implement pre-hybridization at 60 °C for 2 h, and then hybridization at 60 °C for 16 h (the concentration of probe solution is 25 μM, the concentration of working solution is 45 nM). If 300 μL probe solution was added to each sample, 0.54 μL probe + 300 μL pre-hybrid solution is required. Wash twice with 2 × SSC for 10 min each time (wash at hybrid temperature, 60 °C). Wash 3 times with 50% formamide at hybrid temperature of 60 °C, each time 30 min. Wash five times in DPBS, each time for 5 min. Blocking: seal at room temperature for 1 h. The first antibody reaction: the Dig labeled primary antibody (anti-DIG-AP) was diluted with the sealed liquid according to 1:2000, and the reaction was about 16 h overnight at 4 °C. DPBST (0.1% Tween) was washed 5 times, each time for 5 min. AP buffer washed 3 times, each time for 5 min. At room temperature, add NBT/BCIP (Roche, 11697471001, Basle, Swizerland); the duration does not exceed 48 h. Add 400 μL of chromogenic solution to each sample. PBST was washed 3 times, each time for 5 min. Dehydration: 70%, 80%, 95%, 100% (Alcohol gradient). Then, keep in Xylene I, II for 2 min. Neutral gum seal. Observe under a microscope and record pictures.

### 4.14. Intracutaneous Injection in Mice

First, 10 nmol *miR-199a-3p* agomir freeze-dried powder was diluted with 50 μL of RNase free water for intracutaneous injection in mice and cryopreserved every 5 μL. The freeze-dried powder of 50 nmol *miR-199a-3p* antagomir was diluted with 50 μL of RNase free water and frozen every 5 μL. The repackaging method of 10 nmol *miR-199a-3p* agomir negative control dry powder is the same as that of 10 nmol *miR-199a-3p* agomir, and 50 nmol *miR-199a-3p* antagomir negative control dry powder is the same as that of 50 nmol *miR-199a-3p* antagomir. Two litters of mice with the same sex (female) were selected: one litter selected 48-day-old mice (Telogen hair follicle); three mice were from the *miR-199a-3p* agomir group, and another three mice in the same litter were from the *miR-199a-3p* agomir control group. The second litter selected 42-day-old mice (Catagen hair follicle); three mice were from the *miR-199a-3p* antagomir group, while the other three mice were from the *miR-199a-3p* antagomir control group. Every 5 μL of *miR-199a-3p* agomir, *miR-199a-3p* agomir negative control, *miR-199a-3*p antagomir, and *miR-199a-3p* antagomir negative control cryopreservation solution were added with 95 μL of DPBS for each mouse injection. *MiR-199a-3p* agomir group injected *miR-199a-3p* agomir, *miR-199a-3p* agomir control group injected *miR-199a-3p* agomir negative control, *miR-199a-3p* antagomir group injected *miR-199a-3p* antagomir, and *miR-199a-3p* antagomir control group injected *miR-199a-3p* antagomir negative control once a day for 3 days.

### 4.15. Lamb Intravenous Injection

For Lamb intravenous injection experiment, 100 nmol of *miR-199a-3p* agomir and 100 nmol of *miR-199a-3p* agomir negative control were purchased from Guangzhou Ruibo Science and Technology Biotechnology Co., Ltd. (Ribobio, Guangzhou, China). The experimental group included four newborn merino lambs (injected *miR-199a-3p* agomir), and the control group included four newborn merino lambs (injected *miR-199a-3p* agomir negative control). These lambs are of the same sex (female). *MiR-199a-3p* agomir and *miR-199a-3*p agomir negative control were packaged into one tube per 20 nmol and diluted with 1.2 mL of 10% glucose solution, respectively. The lamb should be fixed in a more comfortable position. Standard procedures for jugular vein injection were employed to administer miR-199a-3p agomir and miR-199a-3p agomir negative control solution to the lambs. Each lamb was injected into the jugular vein three times a day, each time with 0.4 mL of diluted *miR-199a-3p* agomir and *miR-199a-3p* agomir negative control solution once every 3 h. The experimental group and the control group were photographed before, and 18 days after injection, and the wool was collected at 0 days, 1 day, 5 days, and 18 days after injection. The venous blood and skin tissue of lambs in the experimental group and control group were collected.

### 4.16. Statistical Analysis

All statistical details for the RNA-seq experiments can be found in the above sections. PCA and heatmap of the mRNA-seq data were performed using the R package (Version 4.2). Venn diagrams of DEGs and DEPs were performed using an online Venn diagram tool (http://bioinformatics.psb.ugent.be/webtools/Venn/, accessed on 24 November 2022). Data were analyzed using two-tailed *t*-tests with the following *p*-values: * *p* < 0.05; ** *p* < 0.01; *** *p* < 0.001.

## 5. Conclusions

By inhibiting the expression of *miR-199a-3p* in vivo, the transcriptional activity of β-catenin was reduced, leading to the development of alopecia-like symptoms in mice. Conversely, by overexpressing *miR-199a-3p* in vivo, the transcriptional activity of β-catenin was increased, thereby promoting the development of local hair follicles in mice. This study thus offers a new animal model for studying the pathogenic mechanisms of alopecia areata.

## Data Availability

The mRNA-seq data reported in this study have been deposited in the National Center for Biotechnology Information database with the accession numbers PRJNA900518 and PRJNA901349.

## Supplementary Materials

The following supporting information can be downloaded at: https://www.mdpi.com/article/10.3390/ijms242417632/s1.
