# Supplementary material for: MiR-199a-3p Regulates the PTPRF/β-Catenin Axis in Hair Follicle Development: Insights into the Pathogenic Mechanism of Alopecia Areata
Source: Int J Mol Sci. 2023 Dec 18;24(24):17632. doi: 10.3390/ijms242417632 (PMC10743674; doi:10.3390/ijms242417632)
Supplement: Supplementary file 1 [file ijms-24-17632-s001.zip › Figure S5.pdf]

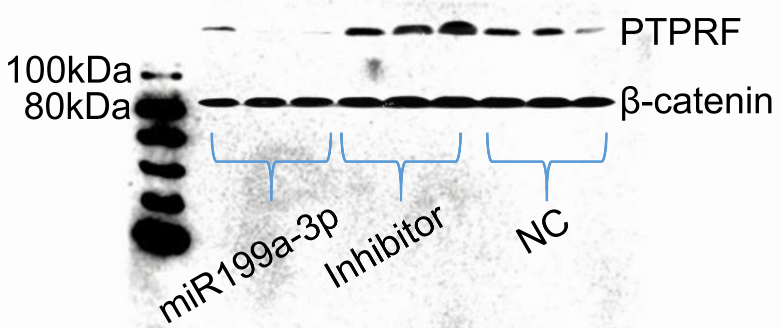

Figure 2C

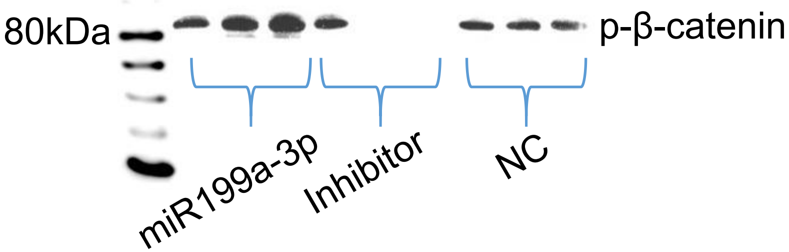

Figure 2C

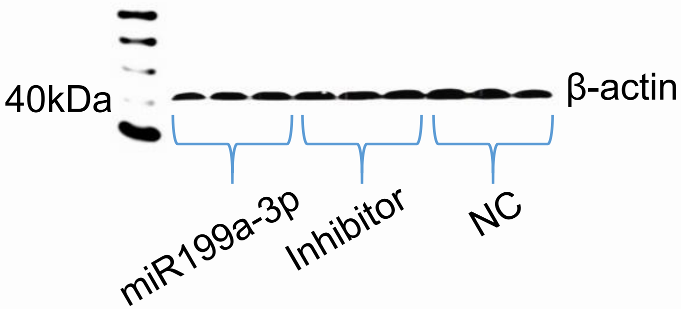

Figure 2C

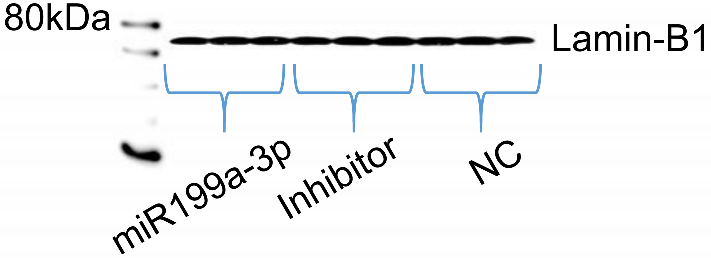

Figure 2E

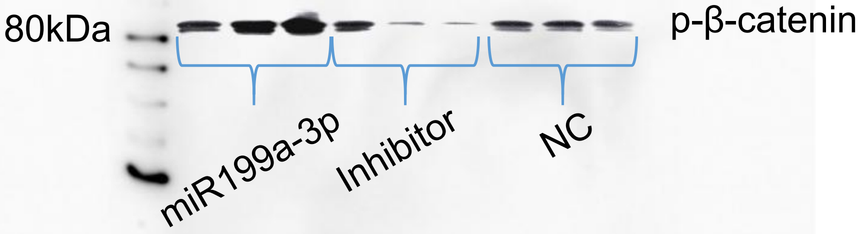

Figure 2E

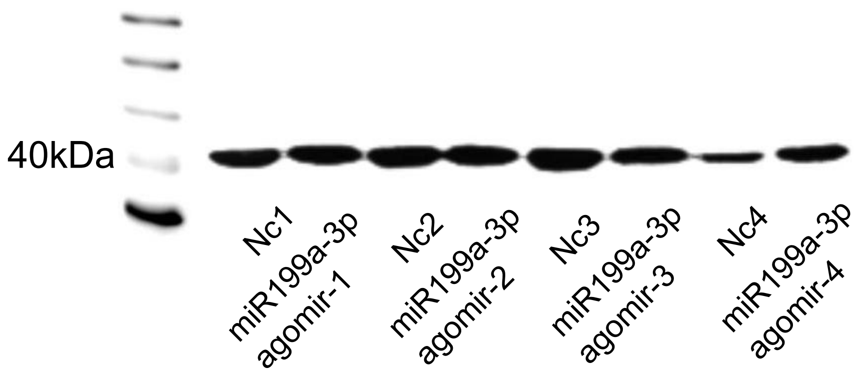

Figure 4C

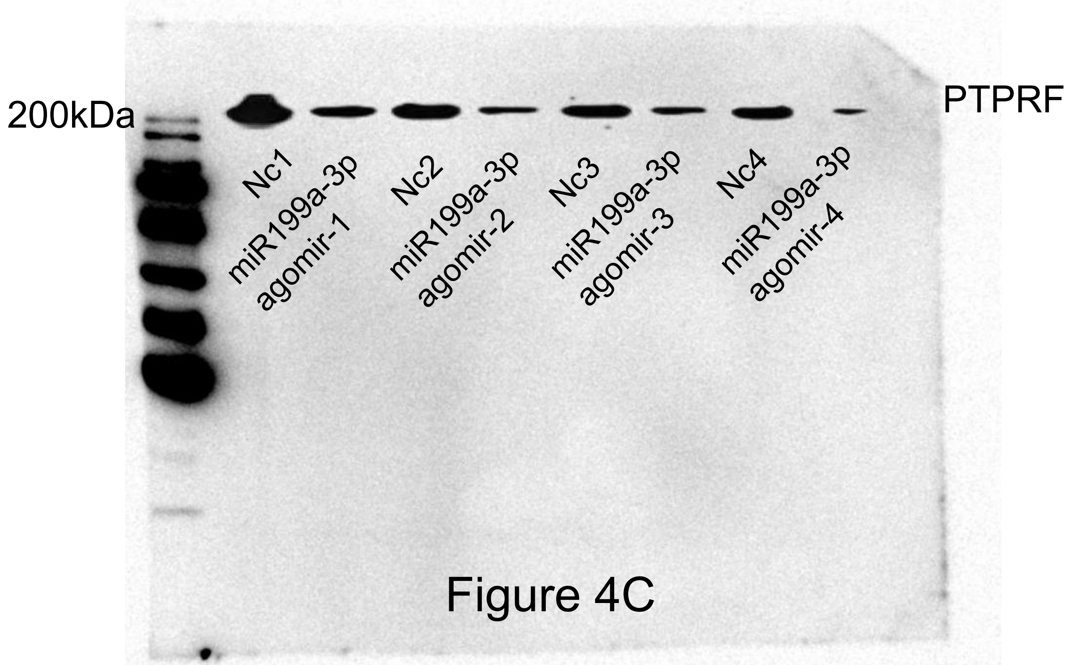

Figure 4C

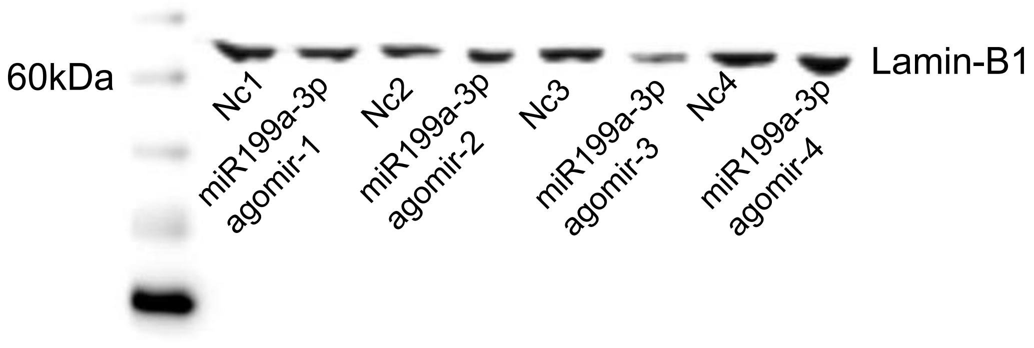

Figure 4D

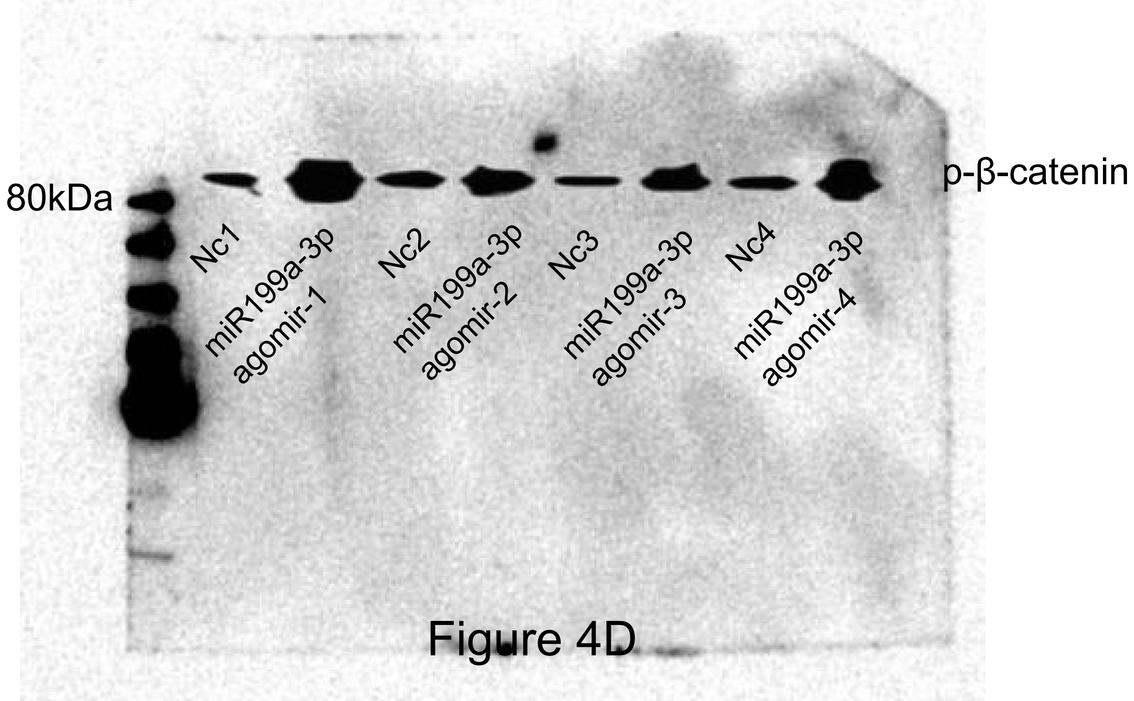

Figure 4D

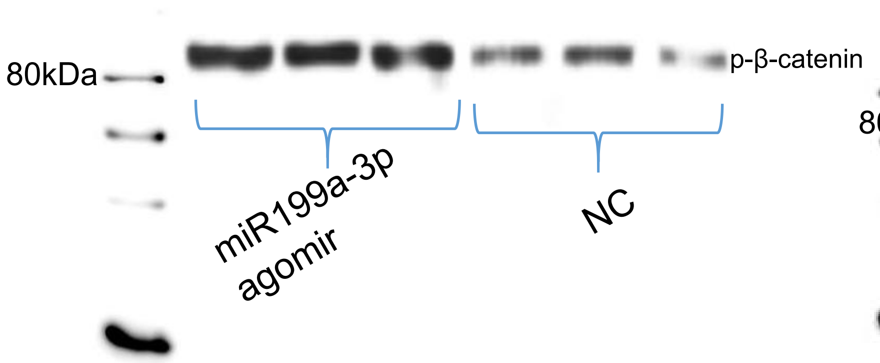

Figure 7A

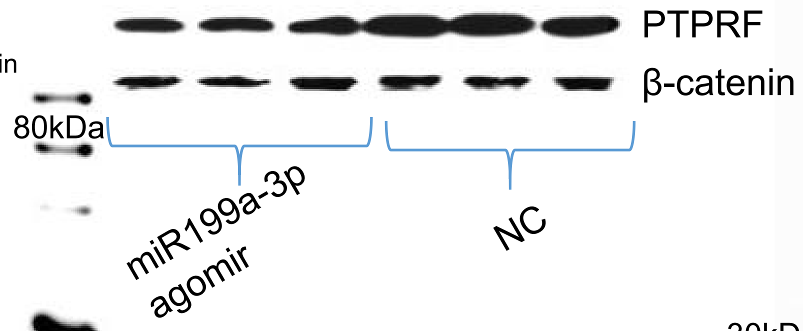

Figure 7A

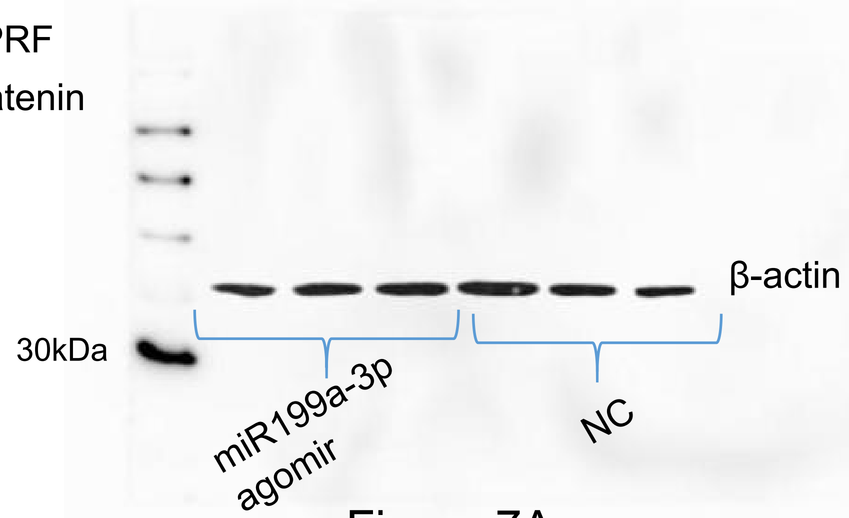

Figure 7A

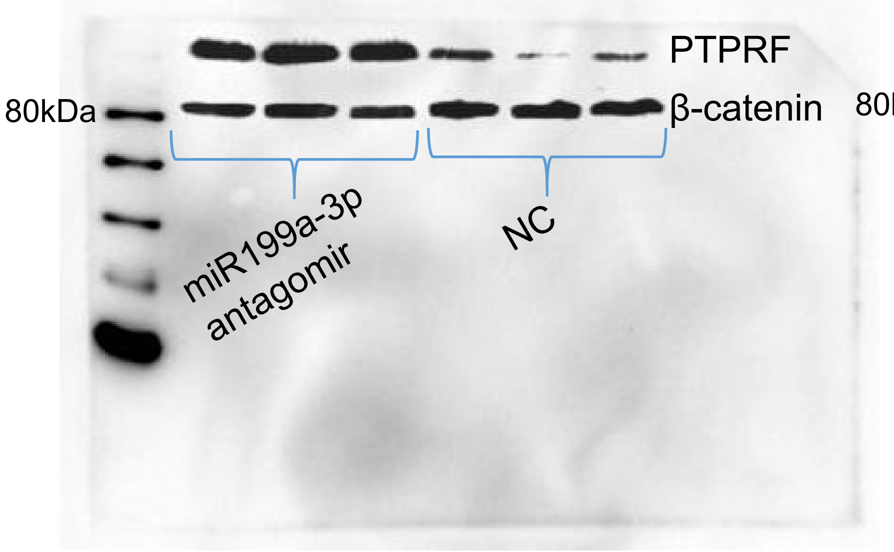

Figure 7B

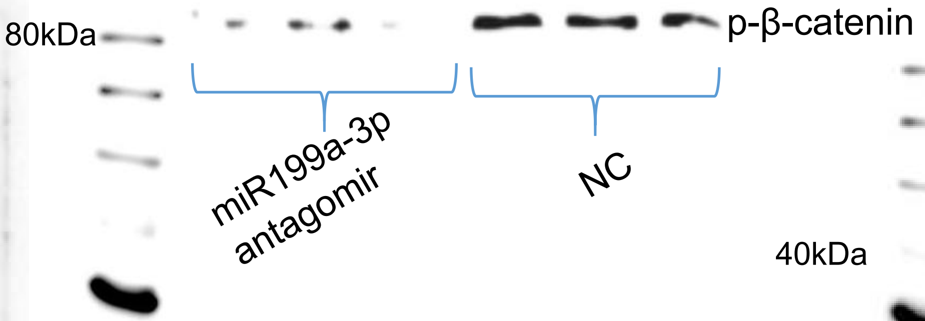

Figure 7B

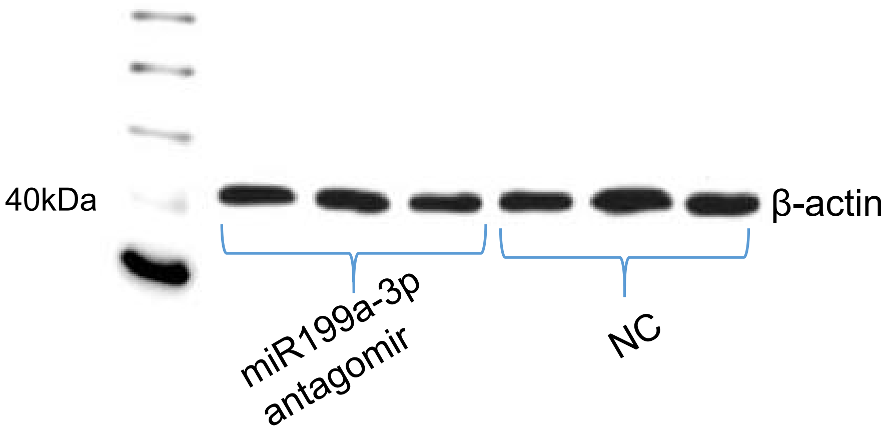

Figure 7B

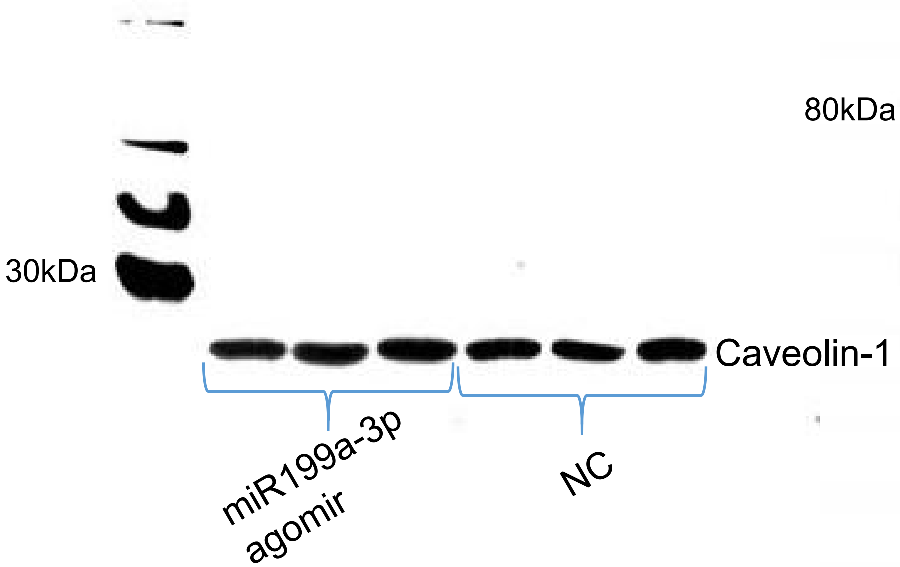

Figure 7E

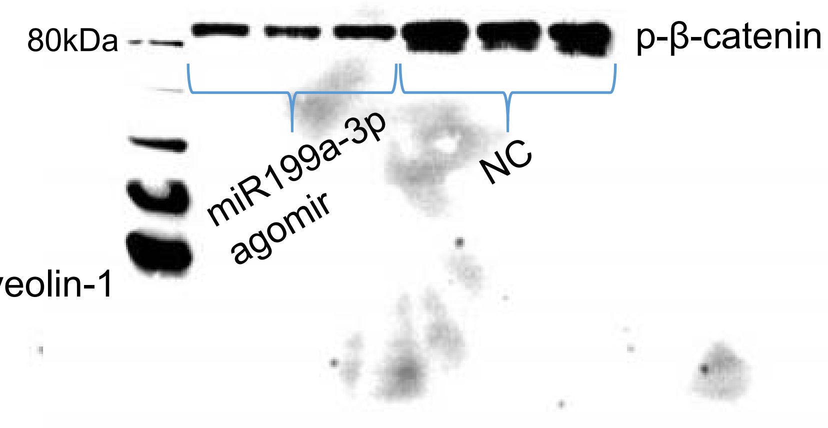

Figure 7E

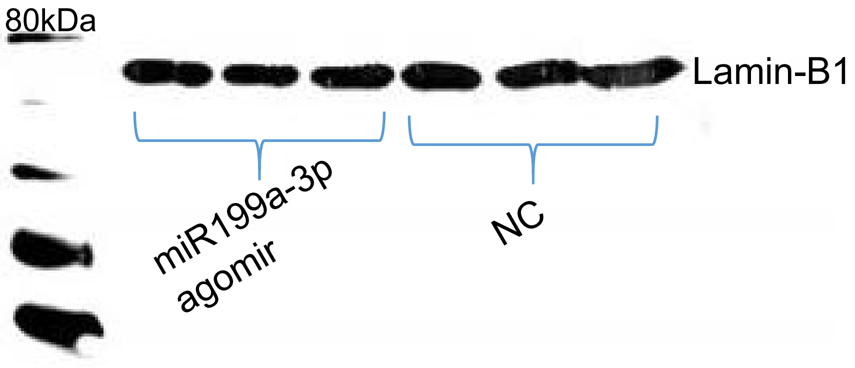

Figure 7F

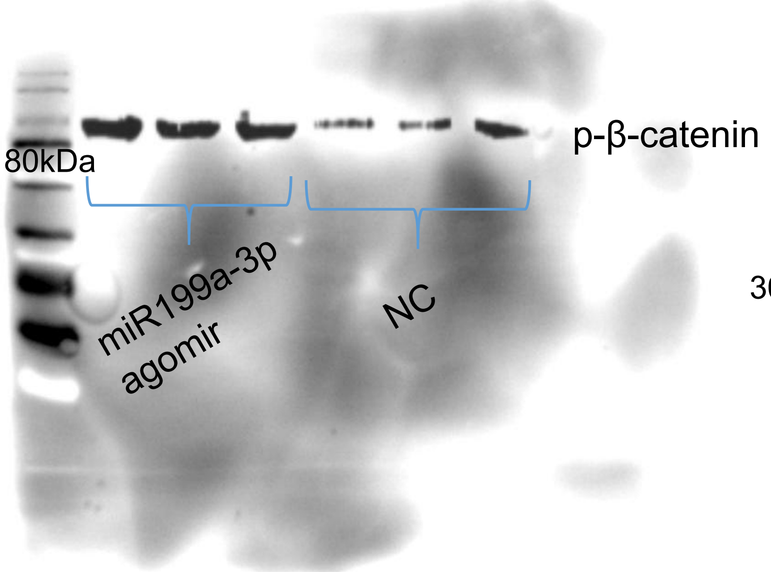

Figure 7F

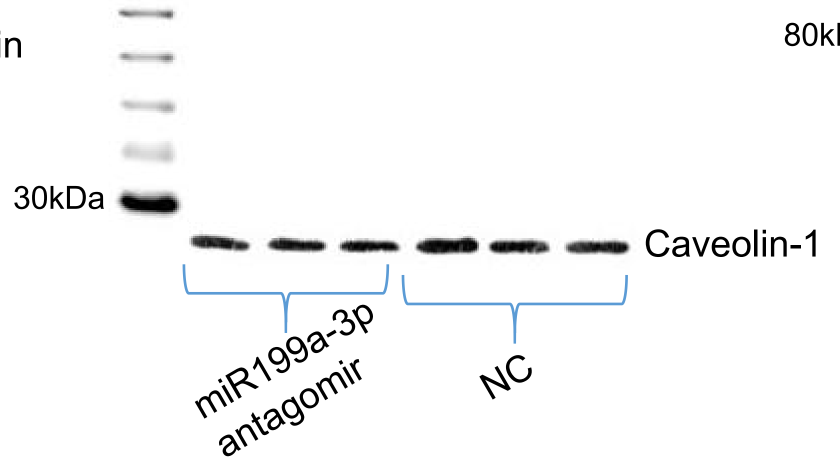

Figure 7I

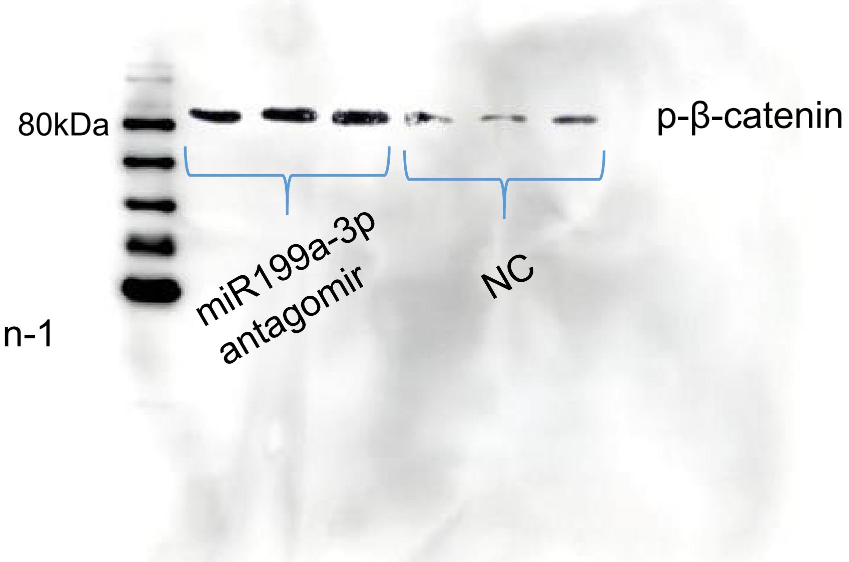

Figure 7I

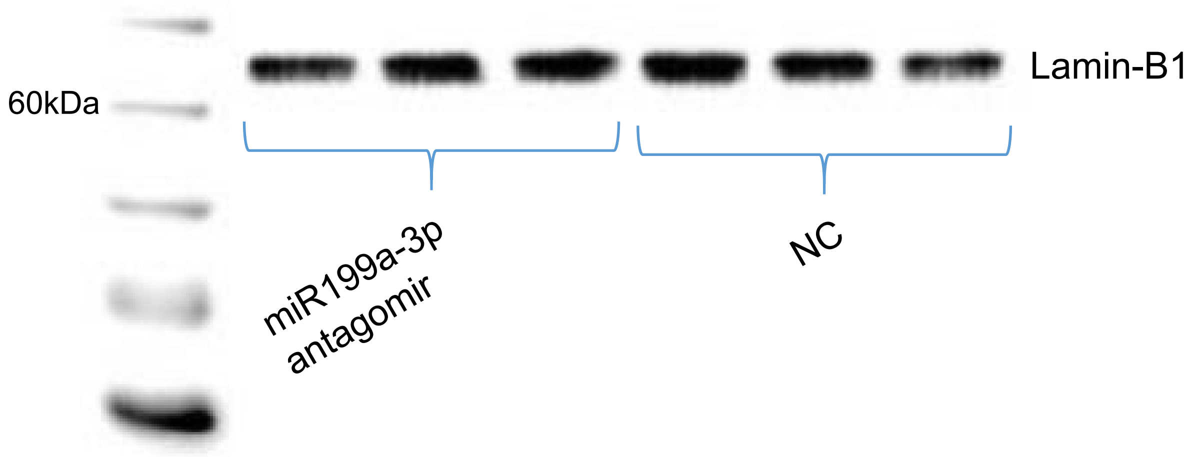

Figure 7J

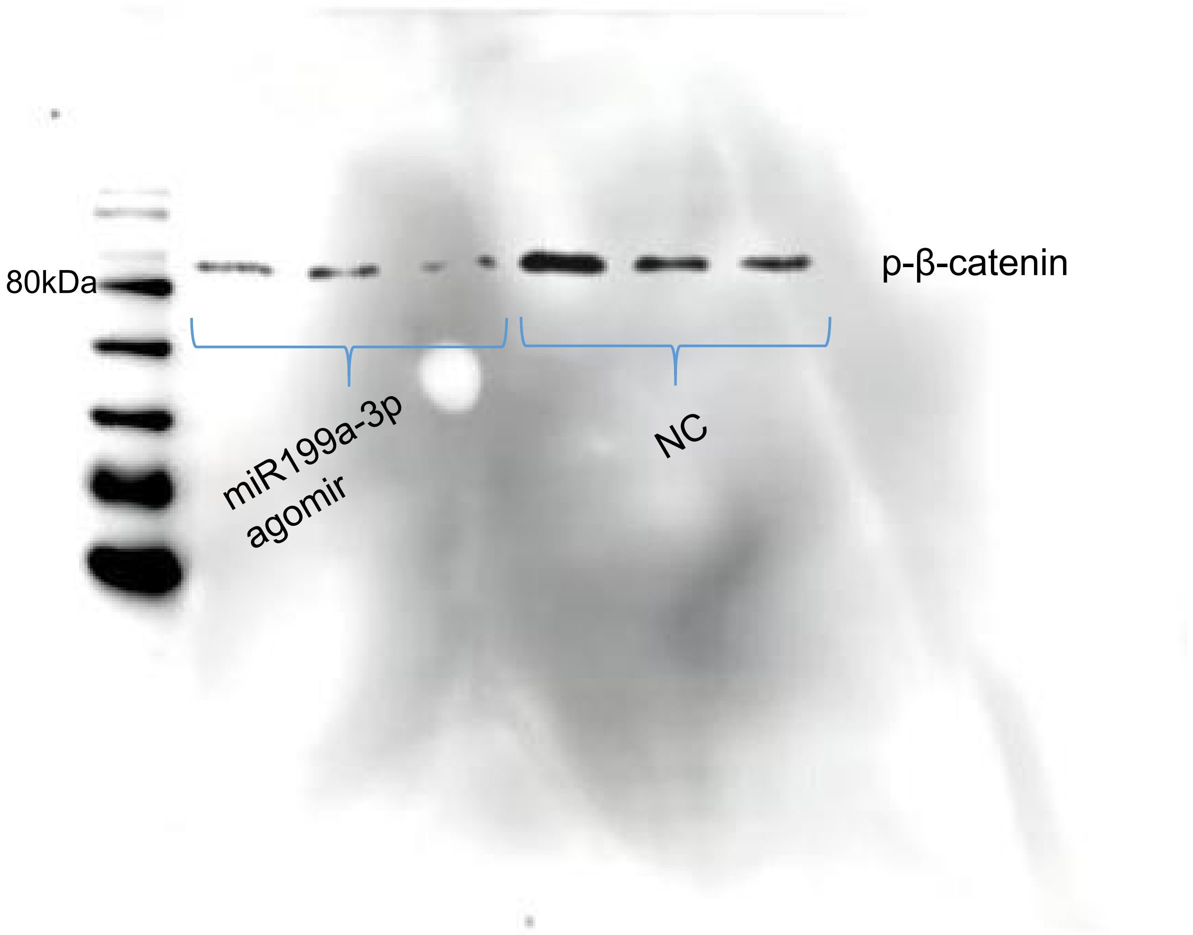

Figure 7J
