# Supplementary material for: MiR-199a-3p Regulates the PTPRF/β-Catenin Axis in Hair Follicle Development: Insights into the Pathogenic Mechanism of Alopecia Areata
Source: Int J Mol Sci. 2023 Dec 18;24(24):17632. doi: 10.3390/ijms242417632 (PMC10743674; doi:10.3390/ijms242417632)
Supplement: Supplementary file 1 [file ijms-24-17632-s001.zip › ijms-2695007-supplementary.pdf]

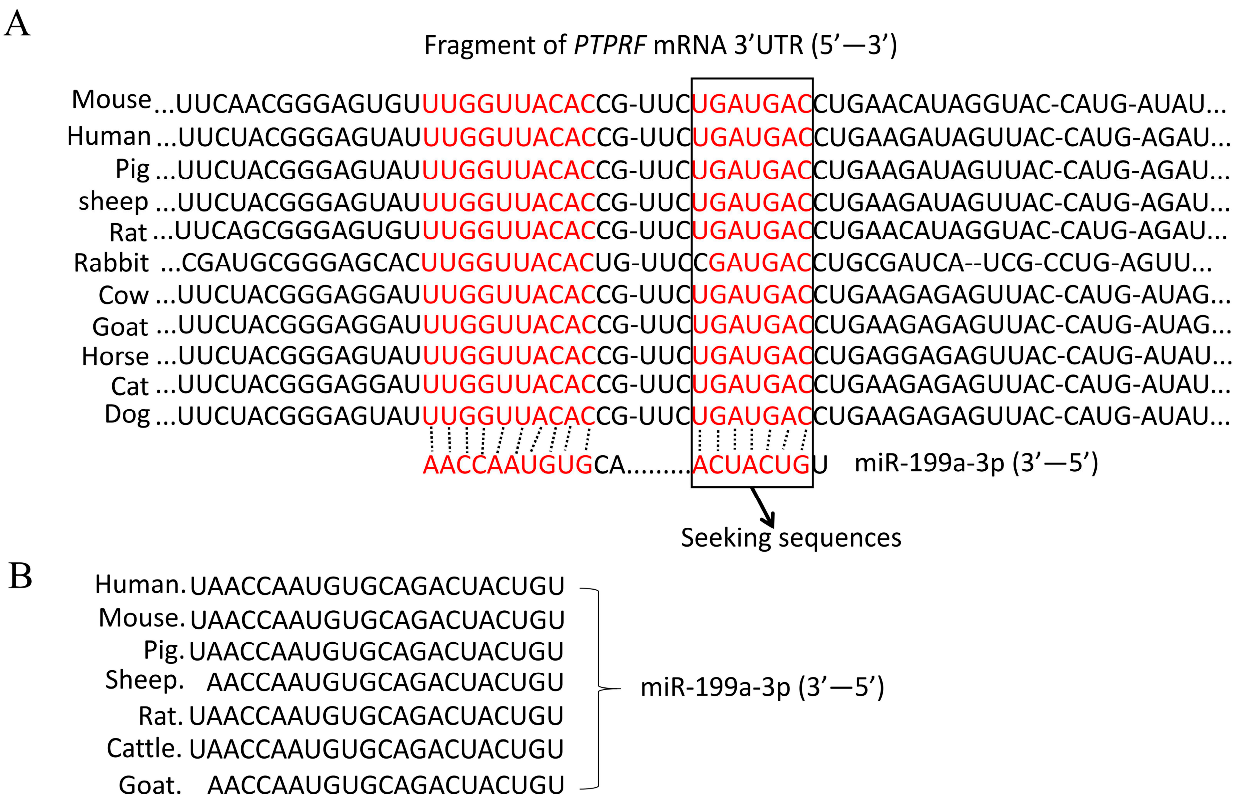

Figure S1: Conservation analysis of *miR-199a-3p* and its target gene PTPRF. (A) Sequence alignment of the PTPRF mRNA 3′ UTR fragment among species. (B) Sequence alignment of *miR-199a-3p* among species.

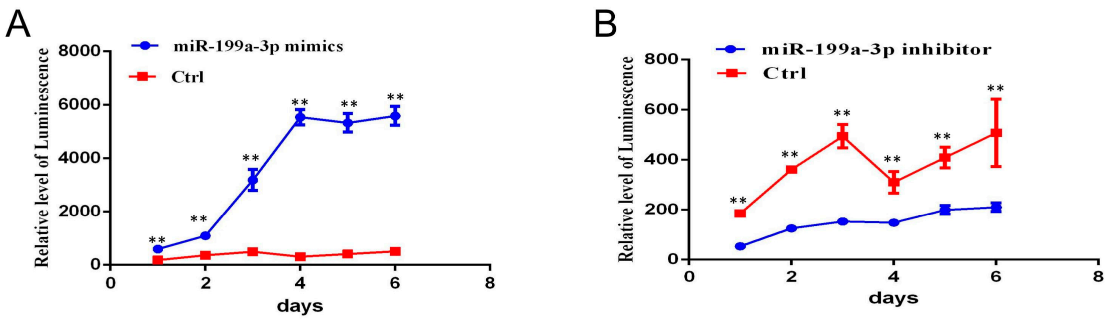

Figure S2: (A) Determination of cell viability after overexpression of *miR-199a-3p* mimics and negative control (** $p < 0.01$), the cell viability was detected by CellTiter–Lumi assay Plus kit (C0068S, Beyotime, Beijing, China). The cell line utilized for cell viability assays was the human 293T cell line, with three experimental replicates conducted at each time point; (B) Determination of cell viability after overexpression of *miR-199a-3p* inhibitors and negative control (** $p < 0.01$). The experimental protocol for cell viability assays, the cell line type employed, and the number of experimental replicates were consistent with Figure S2.

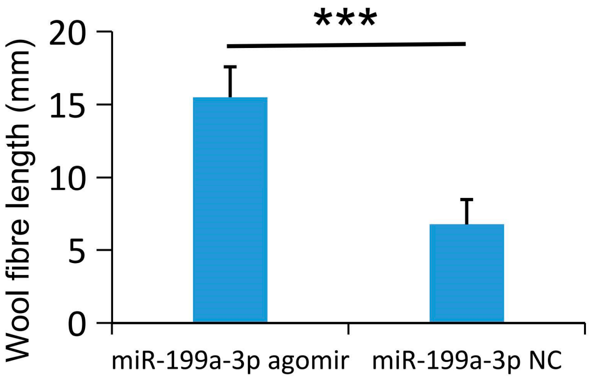

Figure S3: Analysis of wool fiber length after intravenous injection with *miR-199a-3p* agomir and *miR-199a-3p* agomir negative control (*** $p < 0.001$). For lamb intravenous injection experiment, 100 nmol miR-199a-3p agomir and 100 nmol miR-199a-3p agomir negative control were purchased from Guangzhou Ruibo Science and Technology Biotechnology Co., Ltd. (Ribobio, Guangzhou, China). The determination of fiber length involved six 30-day-old female lambs, with the agomir group comprising three lambs and the agomir negative control group comprising three lambs. One hundred wool fibers from the same region of each lamb were selected for the measurement of fiber length.

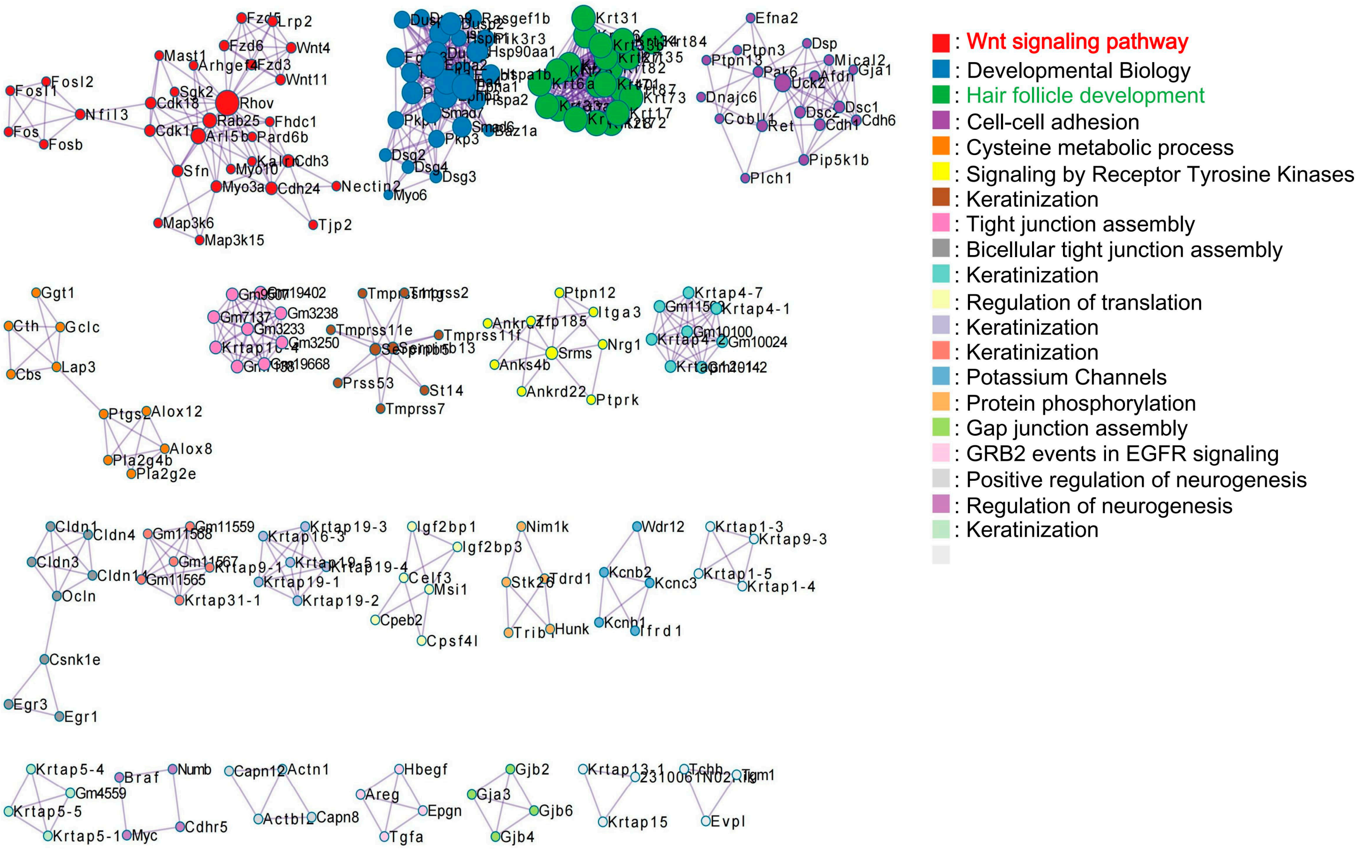

Figure S4: Protein–protein interaction enrichment analysis of the upregulated differentially expressed genes (DEGs) in the IC-agomir group (Metascape online tool; https://metascape.org/gp/index.html).

Figure S5: The full-length uncropped original western blots used in this study. The target proteins were detected by the Gel Documentation System (G: box, SynGene, Cambridge, UK). Band density was analyzed using Image J software.

Table S1: Worksheet 1 contains the primers. Worksheet 2 contains the genome websites.

Table S2. The peptide identification results.

Table S3. The protein quantification and differential expression analysis results in the process of proteomic sequencing.
